# Supplementary figures and images for: Genetic parallelism underpins convergent mimicry coloration in Lepidoptera across 120 million years of evolution
Source: PLoS Biol. 2026 Apr 30;24(4):e3003742. doi: 10.1371/journal.pbio.3003742 (PMC13132254; doi:10.1371/journal.pbio.3003742)

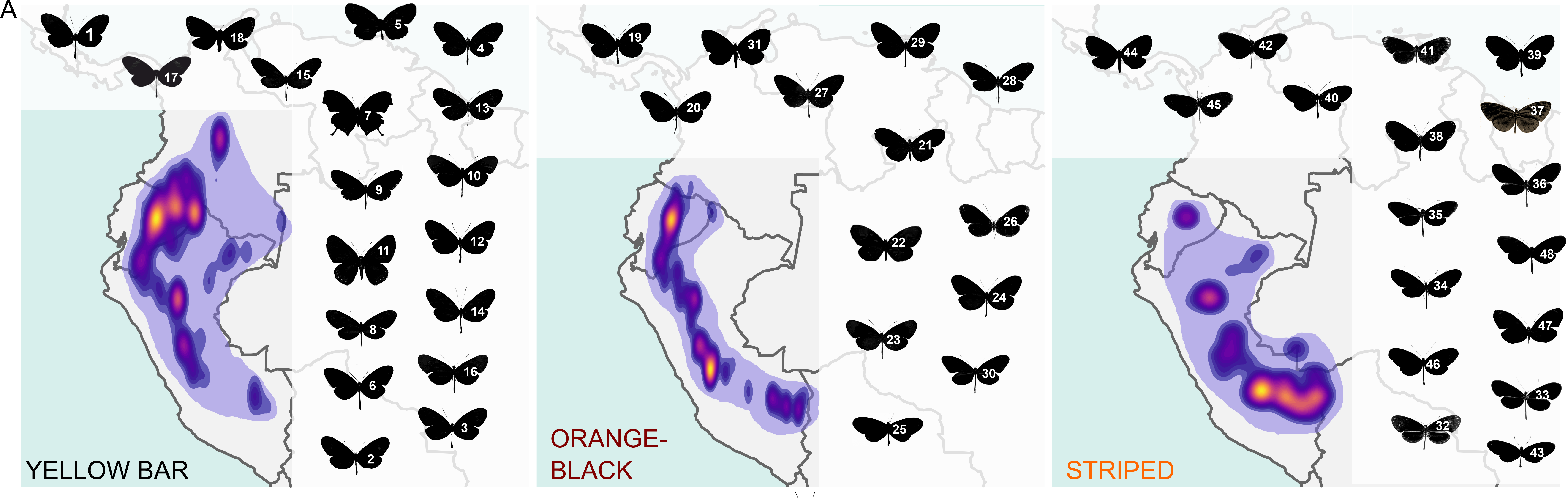

Supplement: S1 Fig — 1). Melinaea menophilus zaneka 2). Hypothyris moebiusi moebiusi 3). Hypothyris mamercus mamercus 4). Heliconius numata euphone 5). Eresia pelonia f. callonia 6). Melinaea isocomma isocomma 7). Consul fabius bogatanus 8). Mechanitis mazeus fallax 9). Forbestra equicola 10). Hypothyris mansuetus amica 11). Themone paid trivittata 12). Hypothyris cantobrica zamorita 13). Hypothyris semifulva ssp. 14). Melinaea mnasias abitagua 15). Mechanitis messenoides messenoides 16). Hypothyris euclea pyrippe 17). Melinaea marseus messenina 18). Chetone histrio histrio 19). Heliconius numata bicoloratus 20). Hypothyris semifulva semifulva 21). Eueides lampeto 22). Eresia pelonia f. Ithomiola 23). Napeogenes rhezia acaea 24). Hypothyris anastasia aureata 25). Hypothyris mansuetus meterus 26). Hyposcada anchiala mendax 27). Melinaea mothone 28). Mechanitis messenoides deceptus 29). Hypothyris anastasia bicolora 30). Melinaea isocomma simulator 31). Chetone histrio hydra 32). Hypothyris euclea callanga 33). Hypothyris fluonia seminigra 34). Forbestra olivencia olivencia 35). Melinaea menophilus orestes 36). Melinaea mnasias romualdo 37). Tithorea harmonia brunnea 38). Napeogenes zurippa deucalion 39). Heliconius pardalinus butleri 40). Melinaea marseus phasiana 41). Hypothyris anastasia anastasina 42). Melinaea menophilus hicetas 43). Mechanitis mazeus mazeus 44). Chetone histrio histrio 45). Hypothyris anastasia acreana 46). Melinaea satevis lamasi 47). Athyrtis mechanitis salvini, and 48). Melinaea marseus clara. Some butterfly images courtesy of https://www.butterfliesofamerica.com/ (Andrew Warren); http://www.sangay.eu/esdex.php/ (Jean-Claude Petit). Maps were generated using openly available base maps from Stadia Maps (stadiamaps.com), with the Stamen Terrain style (stamen.com). (TIF) [file pbio.3003742.s001.tif]

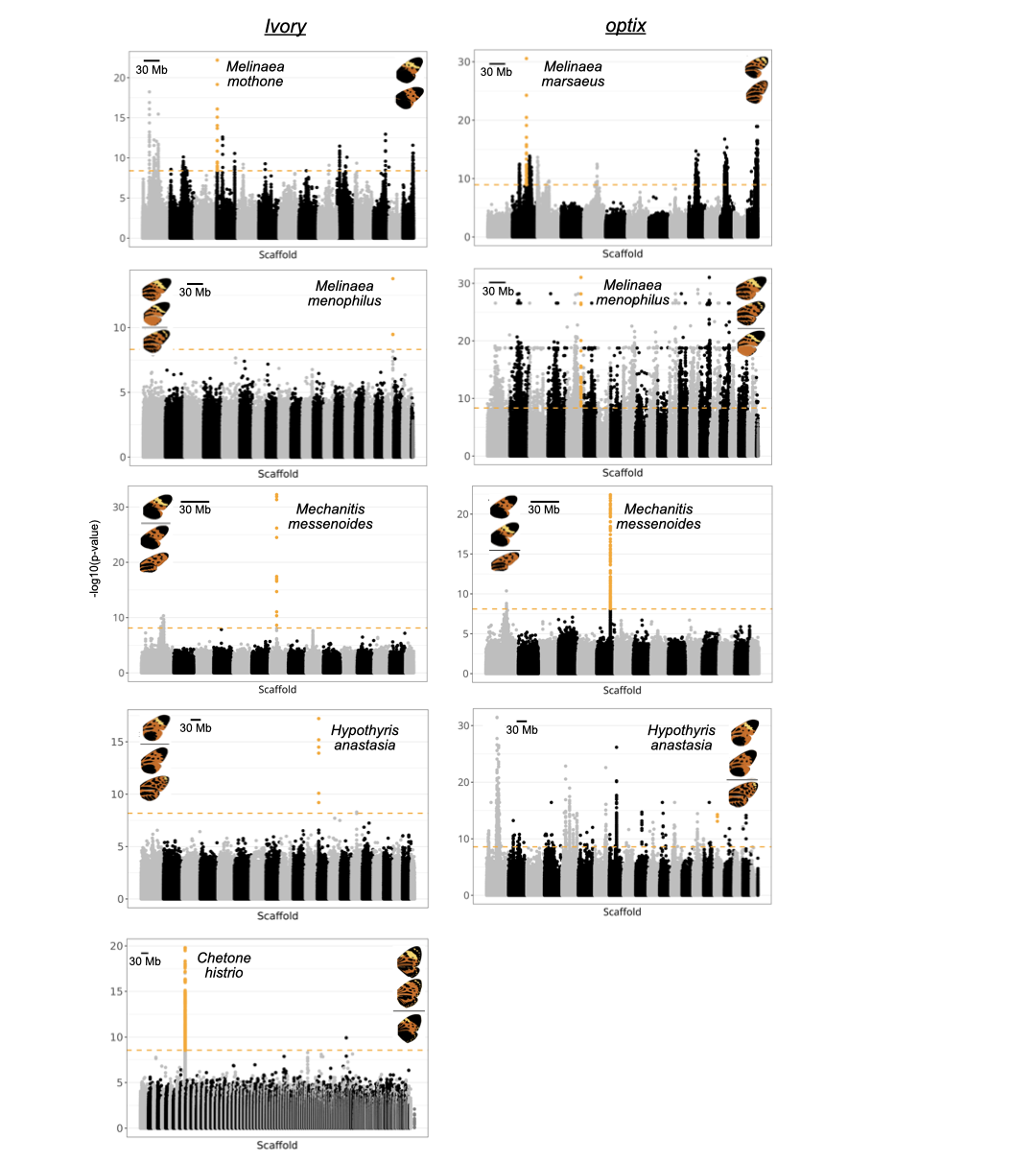

Supplement: S2 Fig — SNPs above the Bonferroni-corrected significance threshold (horizontal dashed line) in the main peak of association are highlighted in orange. The wing images on the right denote the phenotype compared in each analysis. Zoomed-in plots of the peaks are shown in Fig 2. Additional associated SNPs in some of the species are likely a result of population structure correlated with the phenotypes (S17–S20 Figs). The underlying data can be found at https://zenodo.org/records/19135682. (TIFF) [file pbio.3003742.s002.tiff]

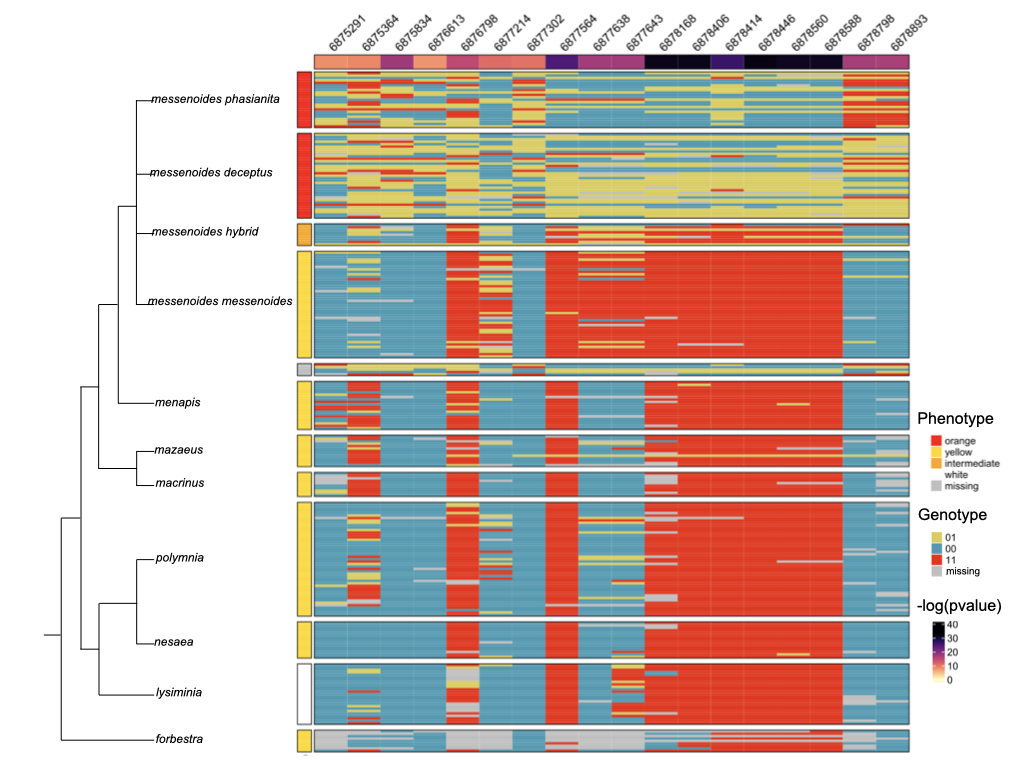

Supplement: S3 Fig — This genotype matrix shows the genotypic states at the top SNPs from the GWA at the ivory locus, with individuals grouped by phylogenetic relationships (left dendrogram). Each row corresponds to an individual and each column to a SNP. The top panels (down to messenoides messenoides) include the focal species used in the GWAS. The remaining taxa are shown to illustrate the lack of association between genotype and phenotype at these SNPs across a broader phylogenetic context. Phenotype group (wing color patterns) is represented by colored boxes to the left of the genotype matrix. The numbers displayed along the top of the figure are the genomic positions of the SNPs. The GWA -log(p-value) values are displayed beneath the genomic positions, with colors ranging from yellow (low significance) to dark purple (high significance). The underlying data can be found at https://zenodo.org/records/19135682. (TIFF) [file pbio.3003742.s003.tiff]

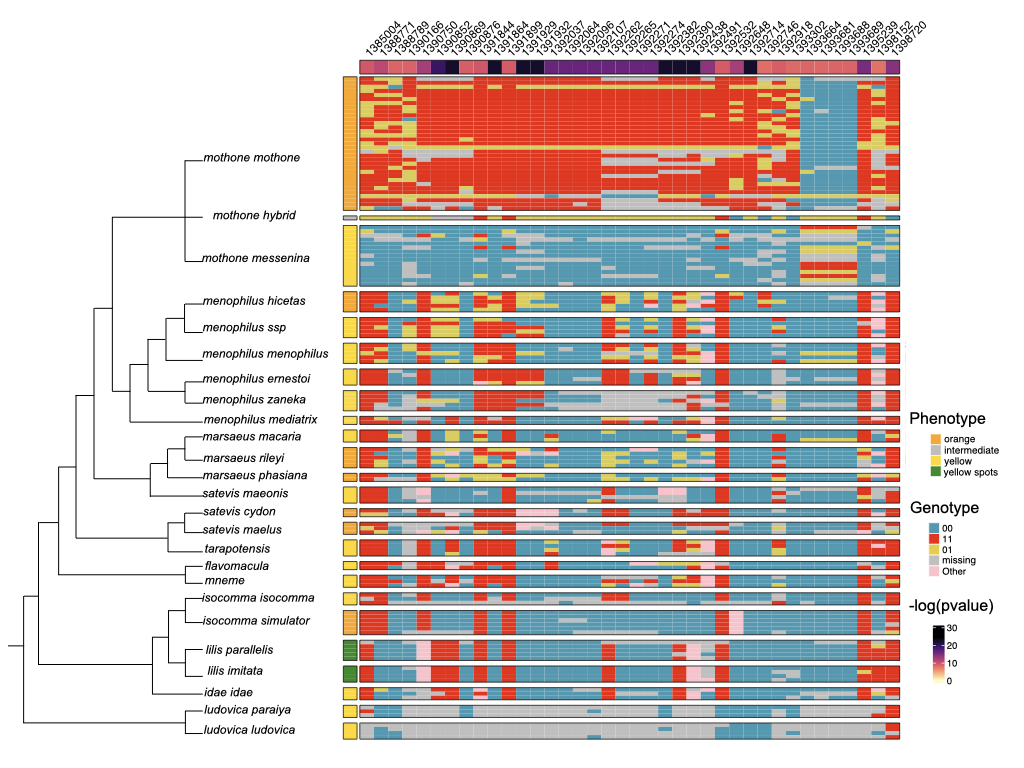

Supplement: S4 Fig — This genotype matrix shows the genotypic states at the top SNPs from the GWA at the ivory locus, with individuals grouped by phylogenetic relationships (left dendrogram). Each row corresponds to an individual and each column to a SNP. The top panels (down to mothone messenina) include the focal species used in the GWAS. The remaining taxa are shown to illustrate the lack of association between genotype and phenotype at these SNPs across a broader phylogenetic context. Phenotype group (wing color patterns) is represented by colored boxes to the left of the genotype matrix. The numbers displayed along the top of the figure are the genomic positions of the SNPs. The GWA -log(p-value) values are displayed beneath the genomic positions, with colors ranging from yellow (low significance) to dark purple (high significance). The underlying data can be found at https://zenodo.org/records/19135682. (TIFF) [file pbio.3003742.s004.tiff]

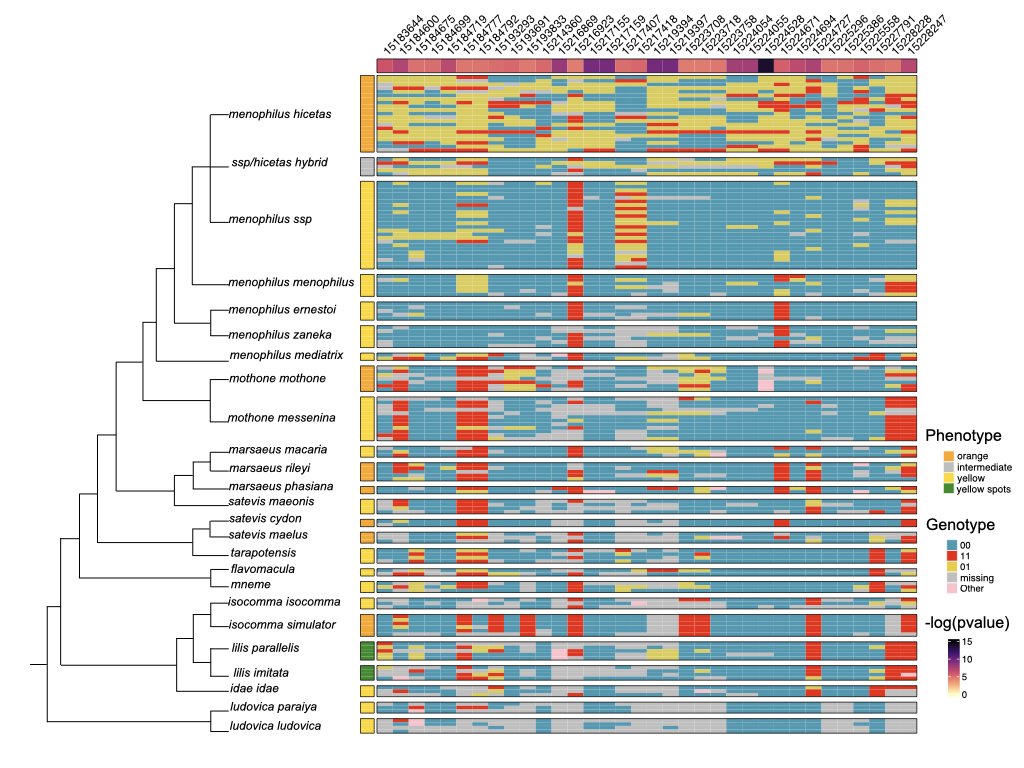

Supplement: S5 Fig — This genotype matrix shows the genotypic states at the top SNPs from the GWA at the ivory locus, with individuals grouped by phylogenetic relationships (left dendrogram). Each row corresponds to an individual and each column to a SNP. The top panels (down to menophilus zaneka) include the focal species used in the GWAS. The remaining taxa are shown to illustrate the lack of association between genotype and phenotype at these SNPs across a broader phylogenetic context. Phenotype group (wing color patterns) is represented by colored boxes to the left of the genotype matrix. The numbers displayed along the top of the figure are the genomic positions of the SNPs. The GWA -log(p-value) values are displayed beneath the genomic positions, with colors ranging from yellow (low significance) to dark purple (high significance). The underlying data can be found at https://zenodo.org/records/19135682. (TIFF) [file pbio.3003742.s005.tiff]

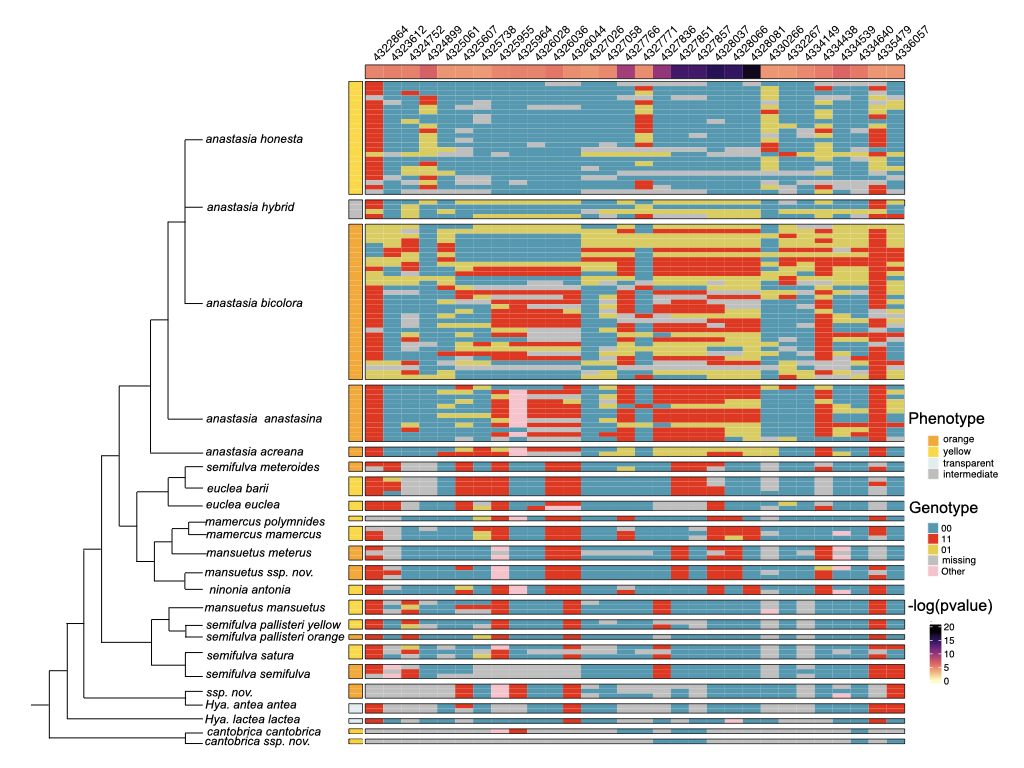

Supplement: S6 Fig — This genotype matrix shows the genotypic states at the top SNPs from the GWA at the ivory locus, with individuals grouped by phylogenetic relationships (left dendrogram). Each row corresponds to an individual and each column to a SNP. The top panels (down to anastasia bicolora) include the focal species used in the GWAS. The remaining taxa are shown to illustrate the lack of association between genotype and phenotype at these SNPs across a broader phylogenetic context. Phenotype group (wing color patterns) is represented by colored boxes to the left of the genotype matrix. The numbers displayed along the top of the figure are the genomic positions of the SNPs. The GWA -log(p-value) values are displayed beneath the genomic positions, with colors ranging from yellow (low significance) to dark purple (high significance). The underlying data can be found at https://zenodo.org/records/19135682. (TIFF) [file pbio.3003742.s006.tiff]

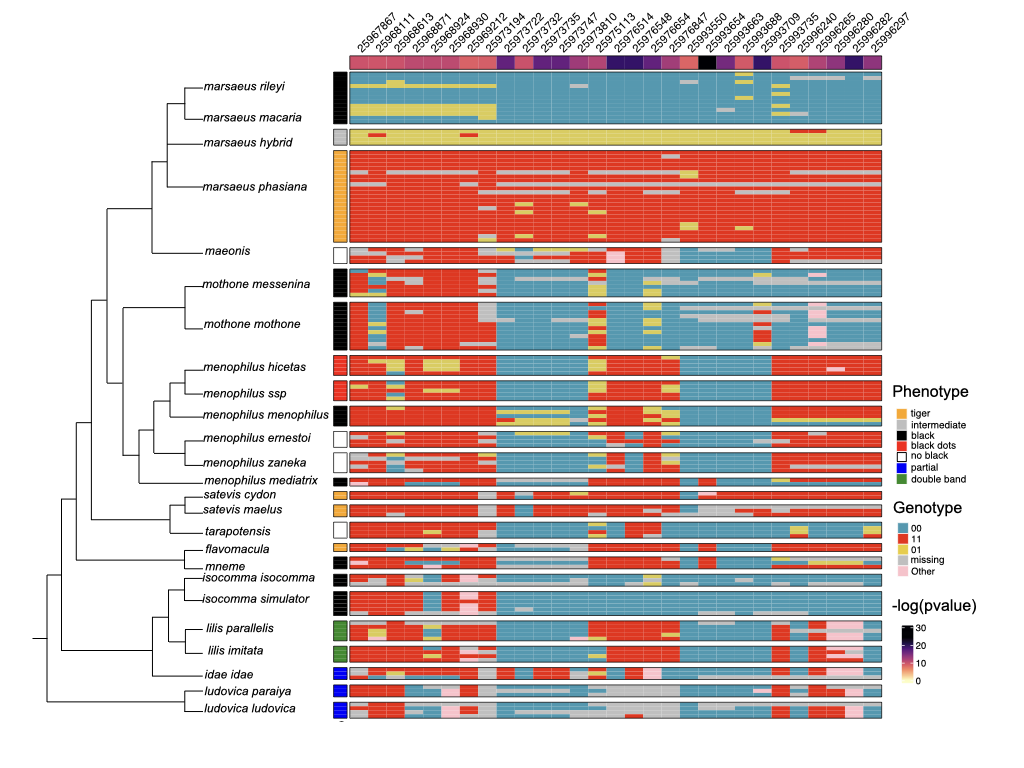

Supplement: S7 Fig — This genotype matrix shows the genotypic states at the top SNPs from the GWA at the optix locus, with individuals grouped by phylogenetic relationships (left dendrogram). Each row corresponds to an individual and each column to a SNP. The top panels (down to marsaeus phasiana) include the focal species used in the GWAS. The remaining taxa are shown to illustrate the association between genotype and phenotype at these SNPs across a broader phylogenetic context. Phenotype group (wing color patterns) is represented by colored boxes to the left of the genotype matrix. The numbers displayed along the top of the figure are the genomic positions of the SNPs. The GWA -log(p-value) values are displayed beneath the genomic positions, with colors ranging from yellow (low significance) to dark purple (high significance). The underlying data can be found at https://zenodo.org/records/19135682. (TIFF) [file pbio.3003742.s007.tiff]

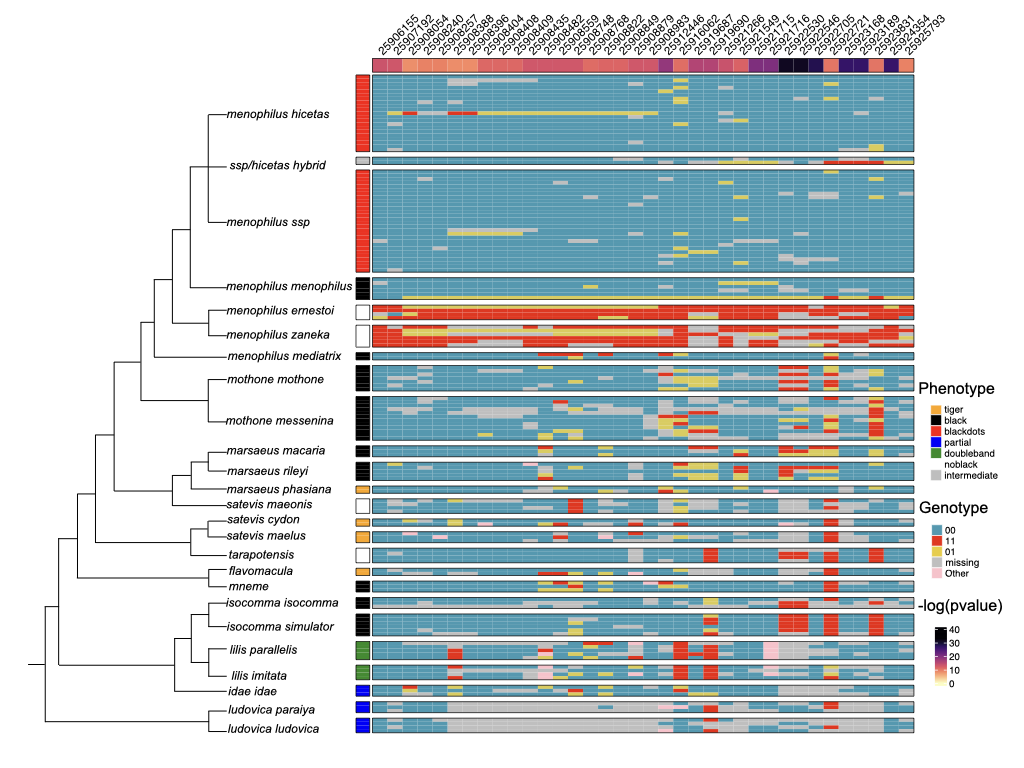

Supplement: S8 Fig — This genotype matrix shows the genotypic states at the top SNPs from the GWA at the optix locus, with individuals grouped by phylogenetic relationships (left dendrogram). Each row corresponds to an individual and each column to a SNP. The top panels (down to menophilus zaneka) include the focal species used in the GWAS. The remaining taxa are shown to illustrate the lack of association between genotype and phenotype at these SNPs across a broader phylogenetic context. Phenotype group (wing color patterns) is represented by colored boxes to the left of the genotype matrix. The numbers displayed along the top of the figure are the genomic positions of the SNPs. The GWA -log(p-value) values are displayed beneath the genomic positions, with colors ranging from yellow (low significance) to dark purple (high significance). The underlying data can be found at https://zenodo.org/records/19135682. (TIFF) [file pbio.3003742.s008.tiff]

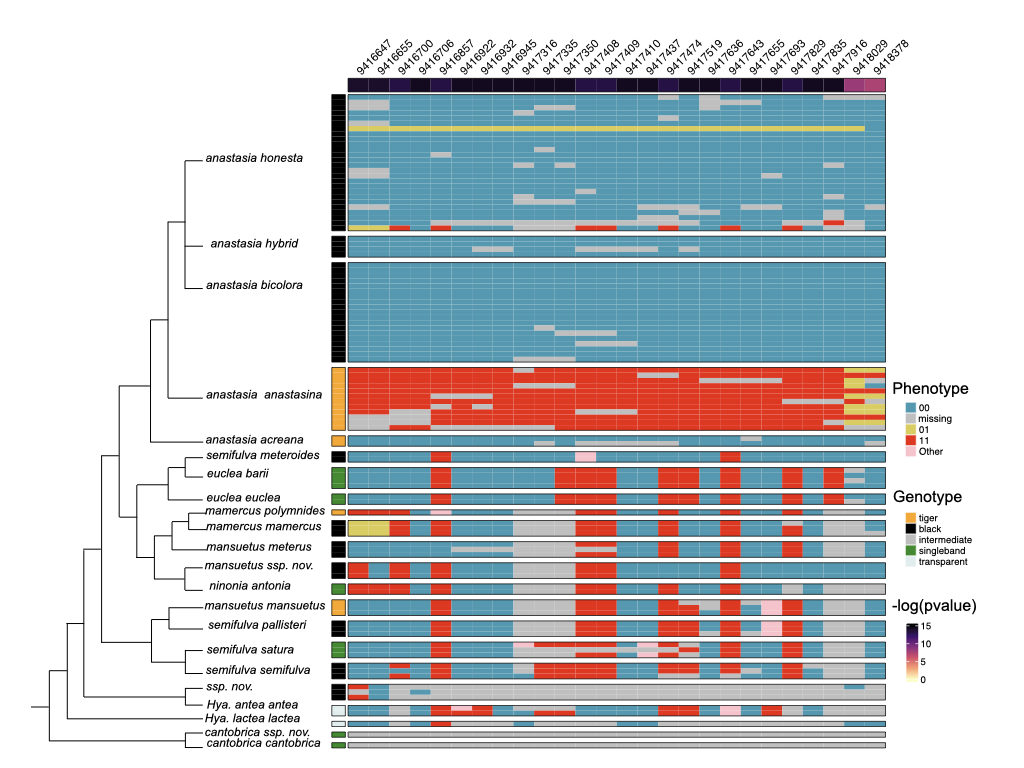

Supplement: S9 Fig — This genotype matrix shows the genotypic states at the top SNPs from the GWA at the optix locus, with individuals grouped by phylogenetic relationships (left dendrogram). Each row corresponds to an individual and each column to a SNP. The top panels (down to anastasia acreana) include the focal species used in the GWAS. The remaining taxa are shown to illustrate the lack of association between genotype and phenotype at these SNPs across a broader phylogenetic context. Phenotype group (wing color patterns) is represented by colored boxes to the left of the genotype matrix. The numbers displayed along the top of the figure are the genomic positions of the SNPs. The GWA -log(p-value) values are displayed beneath the genomic positions, with colors ranging from yellow (low significance) to dark purple (high significance). The underlying data can be found at https://zenodo.org/records/19135682. (TIFF) [file pbio.3003742.s009.tiff]

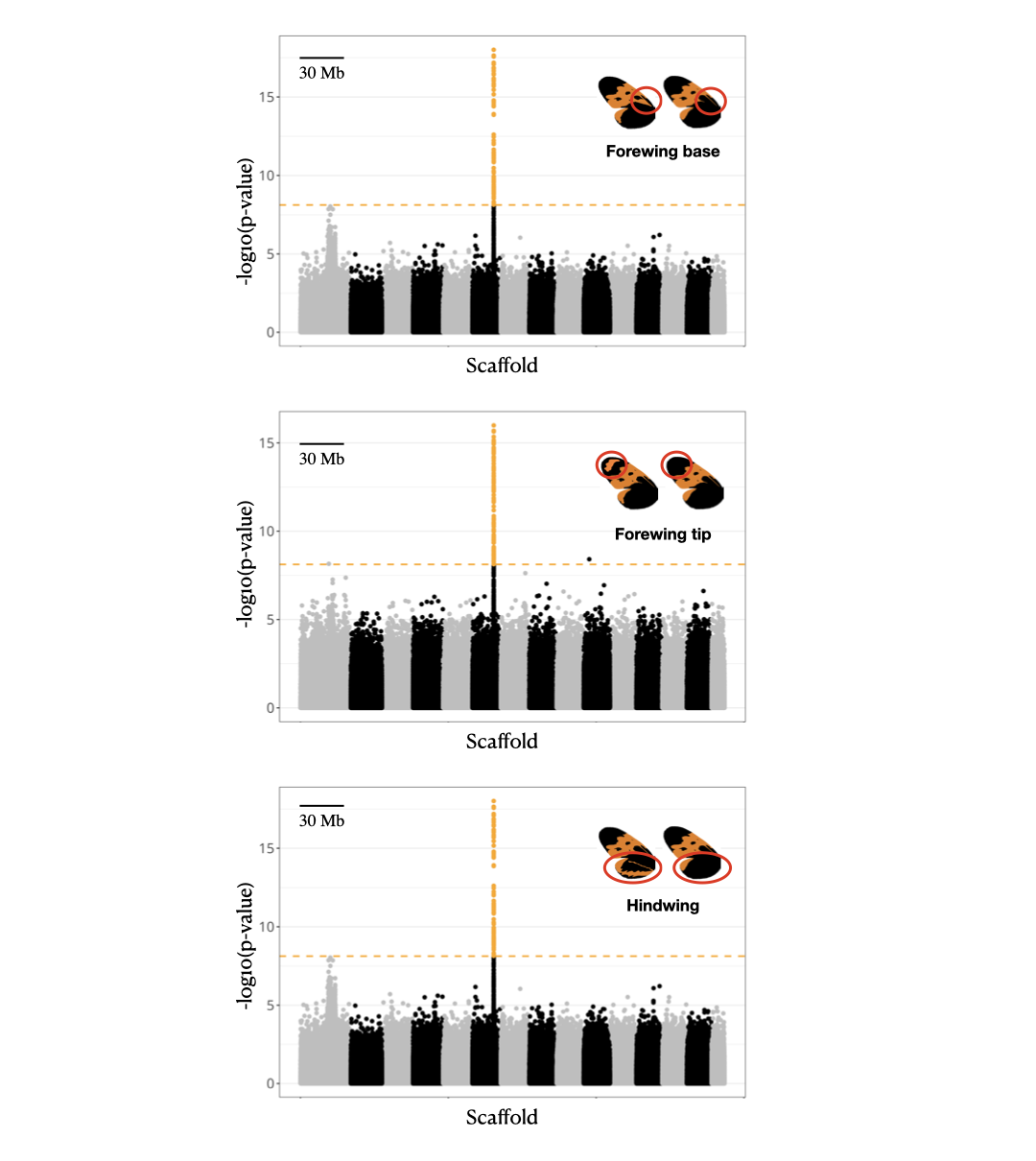

Supplement: S10 Fig — Genome-wide associations for black and orange wing patterns are shown for three different wing regions: forewing base, forewing tip, and hindwing melanization (wing images show the phenotype compared in each analysis). SNPs above the Bonferroni-corrected significance threshold (horizontal dashed line) in the main peak of association are highlighted in orange, which in all cases lies near optix. A zoomed-in plot of the peak is shown in S4A Fig. The underlying data can be found at https://zenodo.org/records/19135682. (TIFF) [file pbio.3003742.s010.tiff]

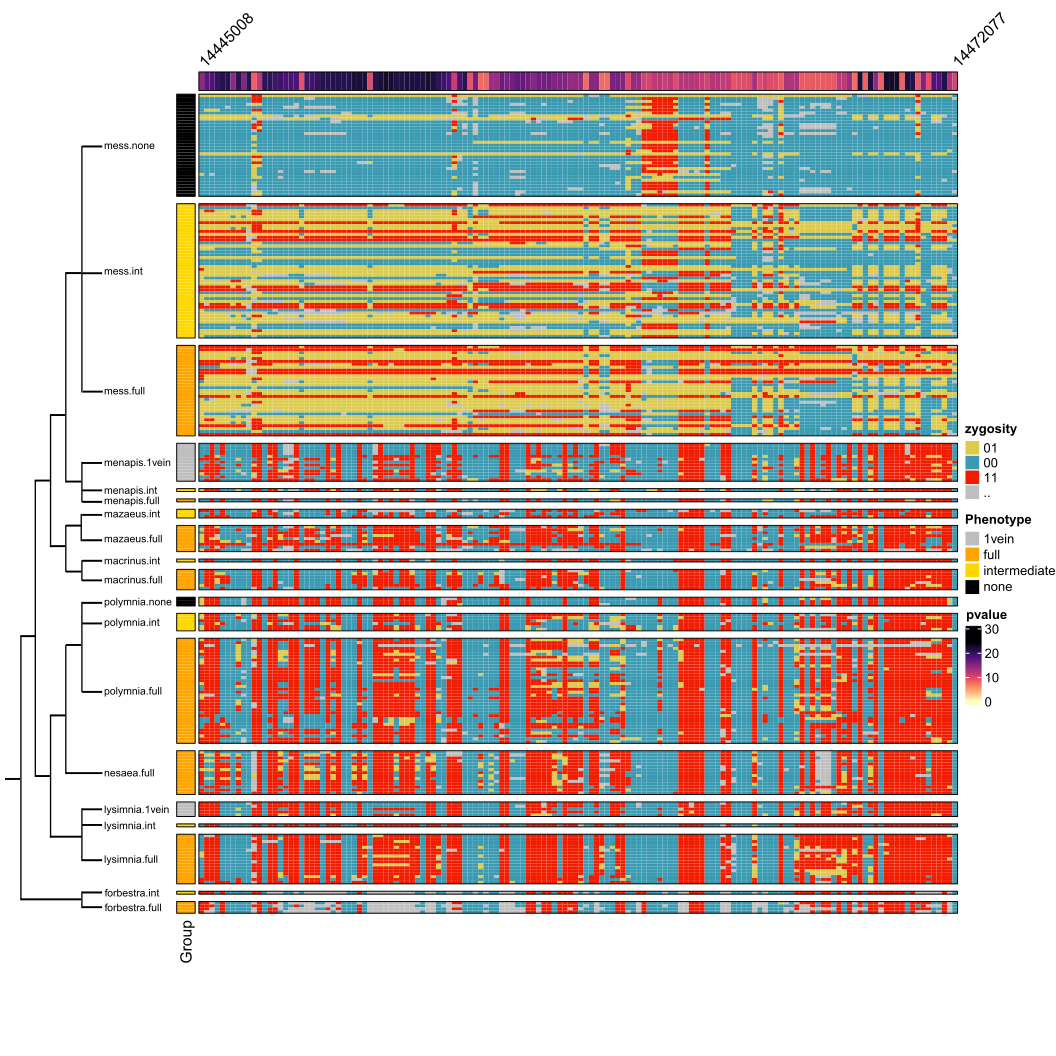

Supplement: S11 Fig — This genotype matrix shows the genotypic states at the top SNPs from the GWA at the optix locus, with individuals grouped by phylogenetic relationships (left dendrogram). Each row corresponds to an individual and each column to a SNP. The top three panels include the focal species used in the GWAS. The remaining taxa are shown to illustrate the lack of association between genotype and phenotype at these SNPs across a broader phylogenetic context. Phenotype group (wing color patterns) is represented by colored boxes to the left of the genotype matrix. The numbers displayed along the top of the figure are the genomic positions of the SNPs. The GWA -log(p-value) values are displayed beneath the genomic positions, with colors ranging from yellow (low significance) to dark purple (high significance). The underlying data can be found at https://zenodo.org/records/19135682. (TIFF) [file pbio.3003742.s011.tiff]

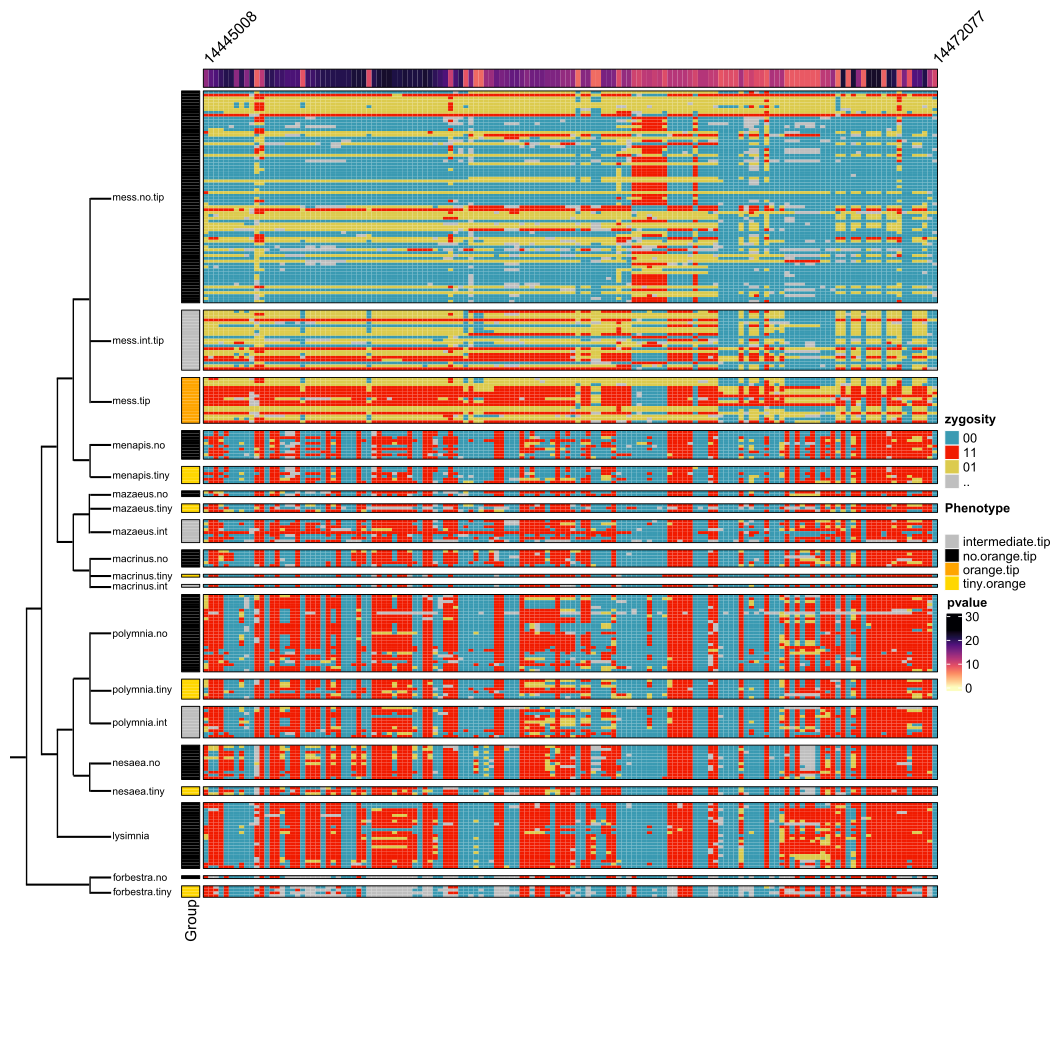

Supplement: S12 Fig — This genotype matrix shows the genotypic states at the top SNPs from the GWA at the optix locus, with individuals grouped by phylogenetic relationships (left dendrogram). Each row corresponds to an individual and each column to a SNP. The top three panels include the focal species used in the GWAS. The remaining taxa are shown to illustrate the lack of association between genotype and phenotype at these SNPs across a broader phylogenetic context. Phenotype group (wing color patterns) is represented by colored boxes to the left of the genotype matrix. The numbers displayed along the top of the figure are the genomic positions of the SNPs. The GWA -log(p-value) values are displayed beneath the genomic positions, with colors ranging from yellow (low significance) to dark purple (high significance). The underlying data can be found at https://zenodo.org/records/19135682. (TIFF) [file pbio.3003742.s012.tiff]

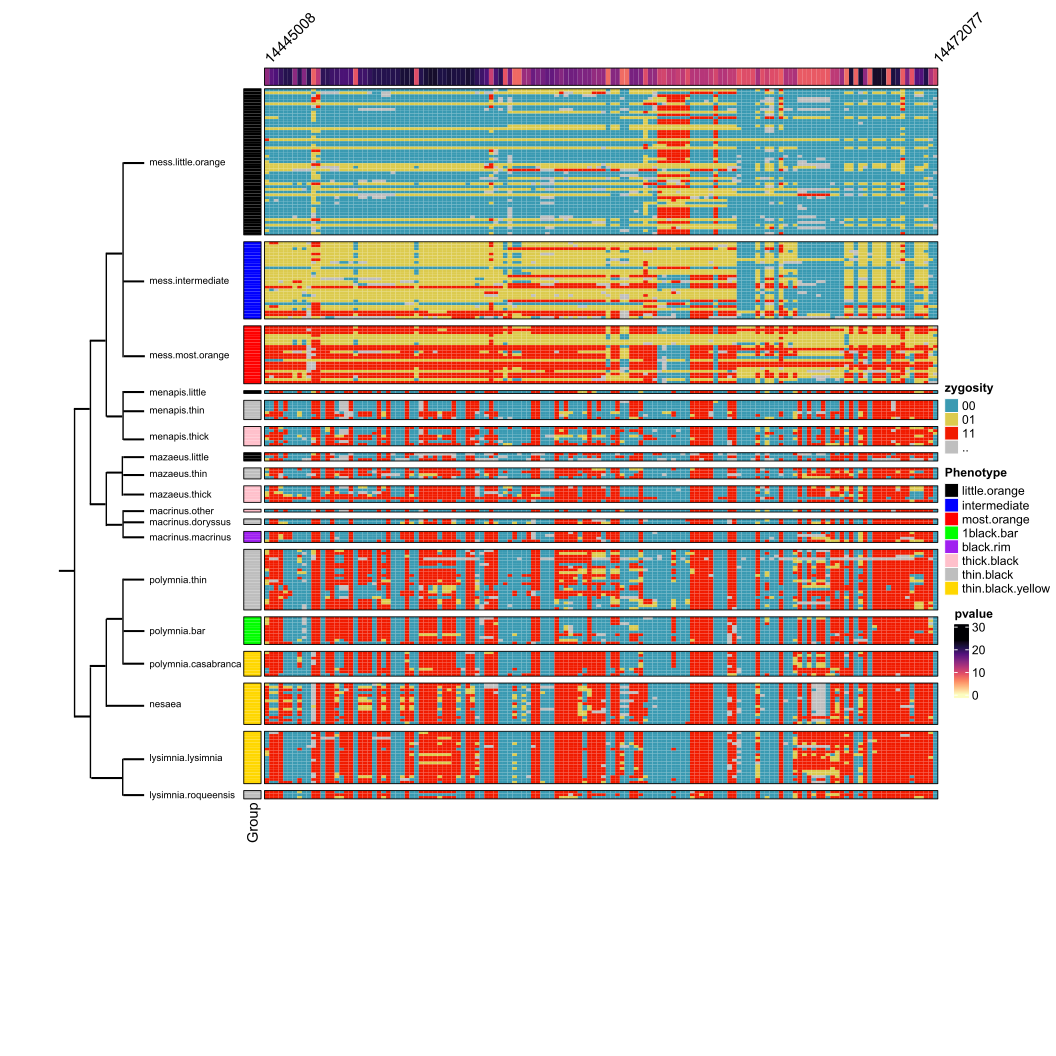

Supplement: S13 Fig — This genotype matrix shows the genotypic states at the top SNPs from the GWA at the optix locus, with individuals grouped by phylogenetic relationships (left dendrogram). Each row corresponds to an individual and each column to a SNP. The top three panels include the focal species used in the GWAS. The remaining taxa are shown to illustrate the lack of association between genotype and phenotype at these SNPs across a broader phylogenetic context. Phenotype group (wing color patterns) is represented by colored boxes to the left of the genotype matrix. The numbers displayed along the top of the figure are the genomic positions of the SNPs. The GWA -log(p-value) values are displayed beneath the genomic positions, with colors ranging from yellow (low significance) to dark purple (high significance). The underlying data can be found at https://zenodo.org/records/19135682. (TIFF) [file pbio.3003742.s013.tiff]

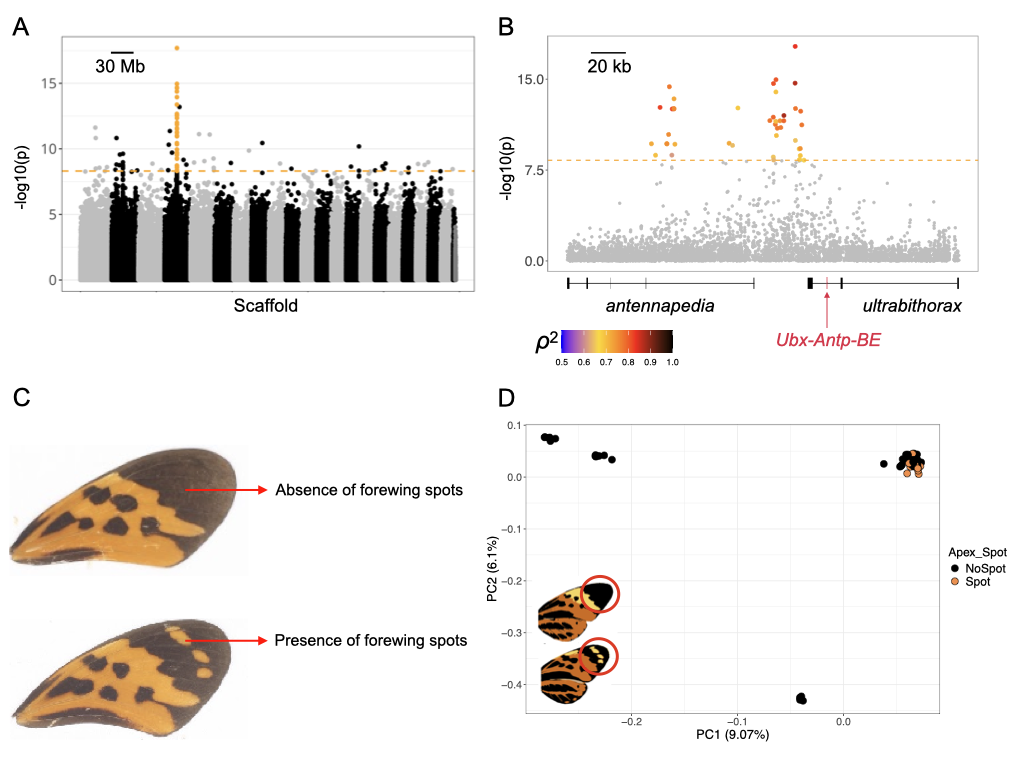

Supplement: S14 Fig — (A) Manhattan plot for the whole-genome. The orange dashed line represents the threshold of significance. SNPs in the main peak of association are highlighted in orange. (B) Zoom on the main peak of association (SUPER_4:16233326–16458737) with locations of annotated genes. SNPs above the Bonferroni-corrected significance threshold (dashed orange line) are colored according to the squared Spearman’s rank correlation coefficient (ρ²), which indicates the strength of association between genotype and phenotype. Red arrow: position of Topologically Associated Domain boundary element Antp-Ubx_BE34. (C) The wing phenotypes compared in the analysis. (D) Principal component analysis of 412,584 LD pruned biallelic SNPs showing the population structure among the sampled individuals. Points are colored by apex spot phenotype. The underlying data can be found at https://zenodo.org/records/19135682. (TIFF) [file pbio.3003742.s014.tiff]

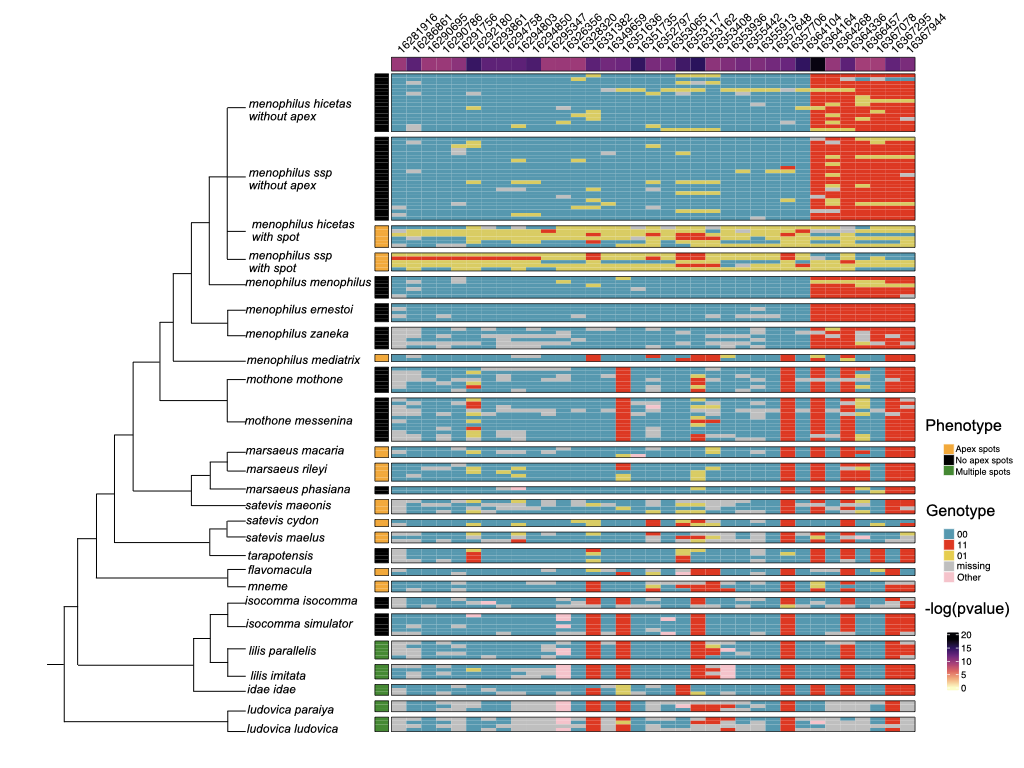

Supplement: S15 Fig — This genotype matrix shows the genotypic states at the top SNPs from the GWA at the antennapedia locus, with individuals grouped by phylogenetic relationships (left dendrogram). Each row corresponds to an individual and each column to a SNP. The top panels (down to menophilus zaneka) include the focal species used in the GWAS. The remaining taxa are shown to illustrate the lack of association between genotype and phenotype at these SNPs across a broader phylogenetic context. Phenotype group (wing color patterns) is represented by colored boxes to the left of the genotype matrix. The numbers displayed along the top of the figure are the genomic positions of the SNPs. The GWA -log(p-value) values are displayed beneath the genomic positions, with colors ranging from yellow (low significance) to dark purple (high significance). The underlying data can be found at https://zenodo.org/records/19135682. (TIFF) [file pbio.3003742.s015.tiff]

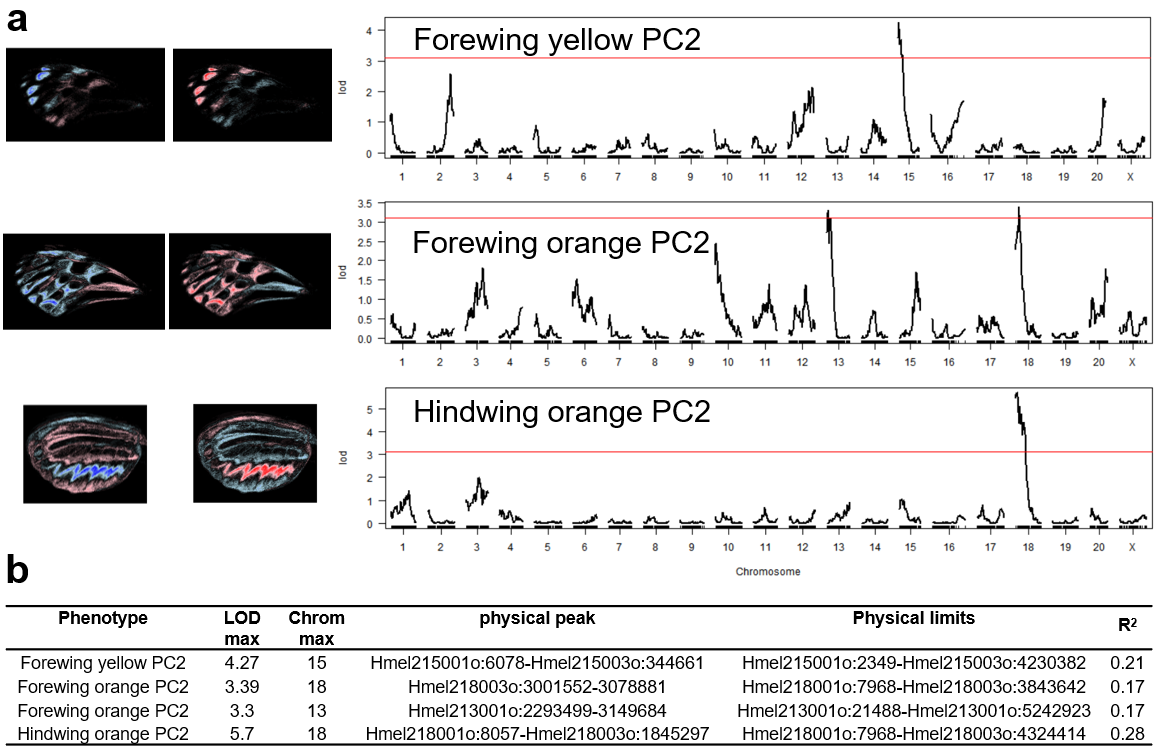

Supplement: S16 Fig — (A) Mapping intervals for yellow and orange forewing and hindwing pattern variation. Heatmap images to the left indicate the phenotypes exhibited by the individuals at the extremes of each PC axis; red = presence of the color, blue = absence of the color. Note that the forewing has only yellow/white, orange/red, and black/brown colors, and the hindwing has only orange/red and black/brown. Since the black/brown pattern variation is a complement of the other colors, we do not show the results of the black/brown variation. (B) Details of the QTL mapping intervals. The underlying data can be found at https://zenodo.org/records/19135682. (TIFF) [file pbio.3003742.s016.tiff]

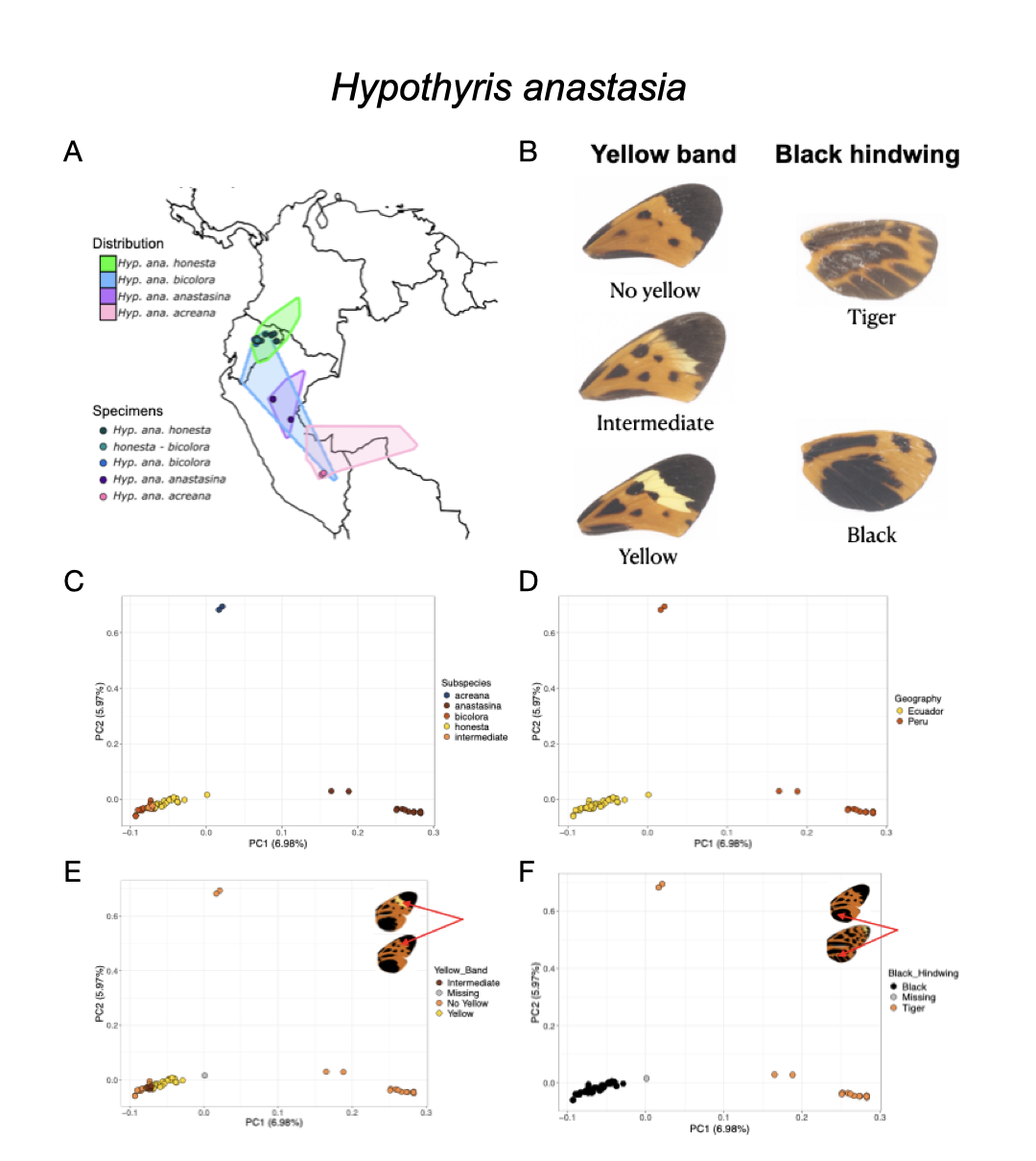

Supplement: S17 Fig — (A) Geographical distribution of Hypothyris anastasia subspecies, with specimen collection locations indicated. Map produced using Natural Earth base layer (https://www.naturalearthdata.com/downloads/50m-cultural-vectors/50m-admin-0-countries-2/) (B) Phenotypes used in the GWA. (C to F): Principal component analysis (PCA) showing the genetic distance between the sampled Hypothyris anastasia individuals using a dataset consisting of 400,452 LD pruned biallelic SNPS. The scatter plots correspond to the first two principal components (PCs). Points are colored by subspecies (C), geography (D), yellow band phenotype (E), and hindwing black phenotype (D). The wing images highlight the color-coded phenotypes. The underlying data can be found at https://zenodo.org/records/19135682. (TIFF) [file pbio.3003742.s017.tiff]

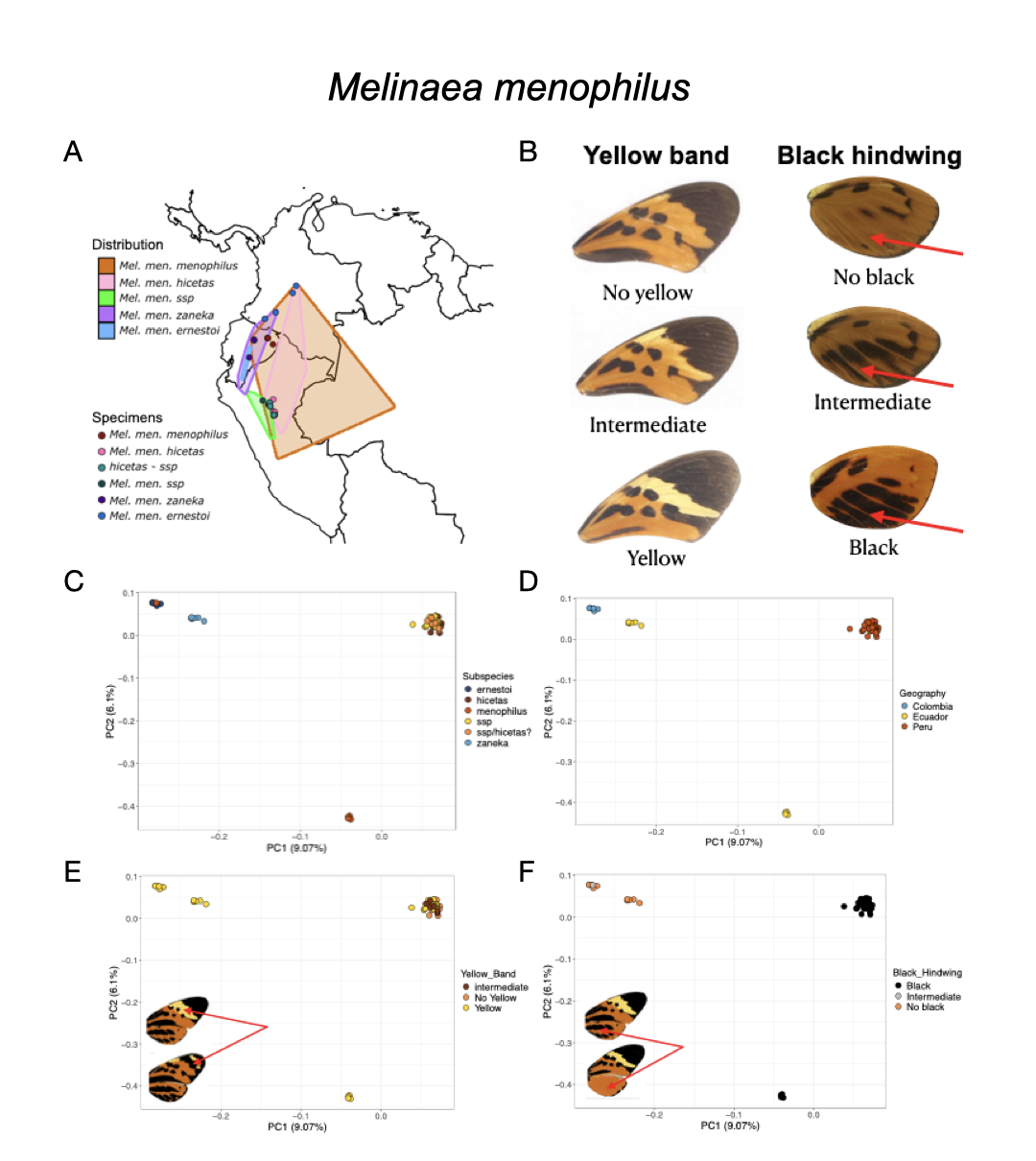

Supplement: S18 Fig — (A) Geographical distribution of Melinaea menophilus subspecies, with specimen collection locations indicated. Map produced using Natural Earth base layer (https://www.naturalearthdata.com/downloads/50m-cultural-vectors/50m-admin-0-countries-2/) (B) Phenotypes used in the GWA. (C to F): Principal Principal component analysis (PCA) showing the genetic distance between the sampled Melinaea menophilus individuals using a dataset consisting of 412,584 LD pruned biallelic SNPS. The scatter plots correspond to the first two principal components (PCs). Points are colored by subspecies (C), geography (D), yellow band phenotype (E), and hindwing black phenotype (F). The wing images highlight the color-coded phenotypes. The underlying data can be found at https://zenodo.org/records/19135682. (TIFF) [file pbio.3003742.s018.tiff]

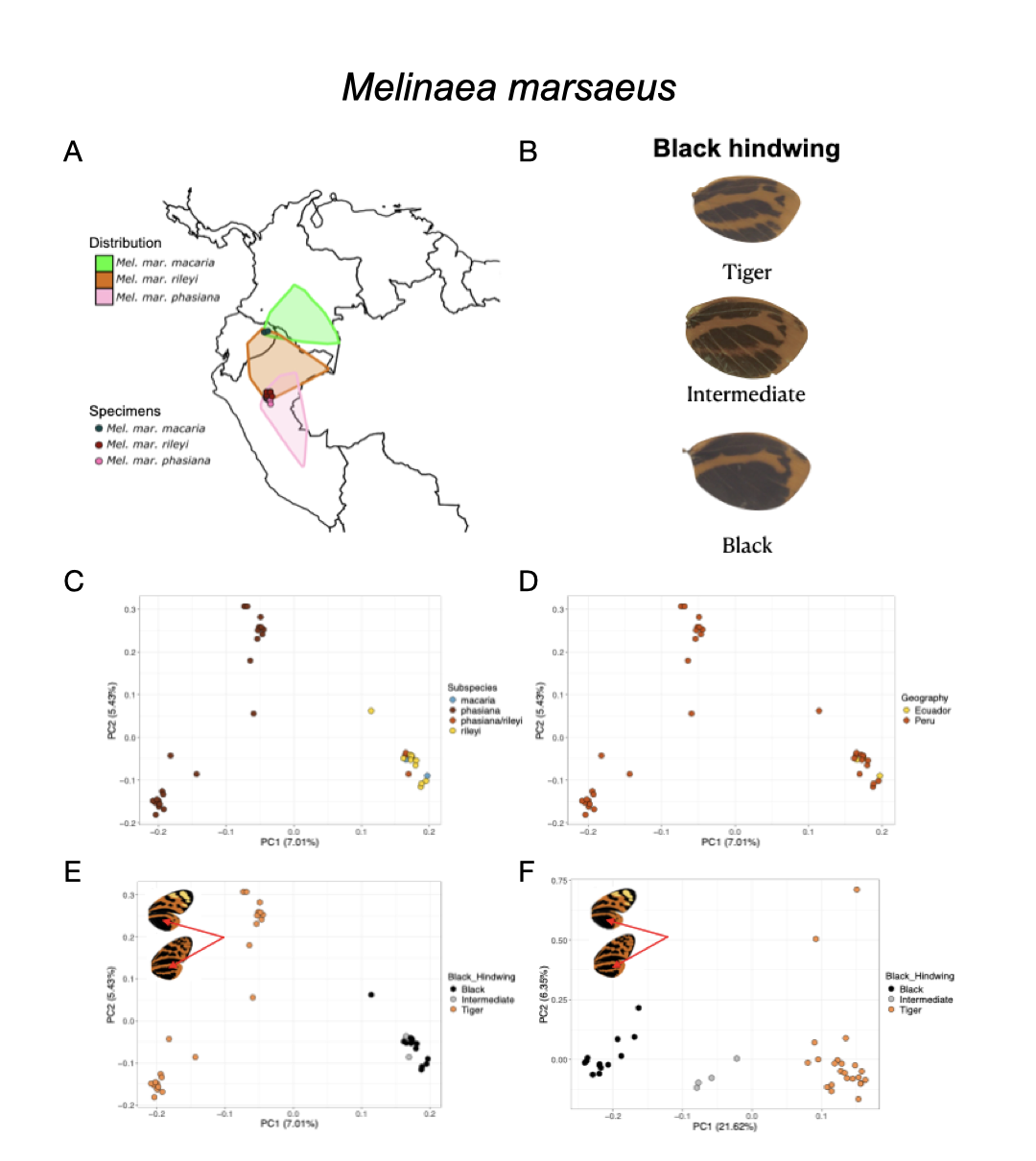

Supplement: S19 Fig — (A) Geographical distribution of Melinaea marsaeus subspecies, with specimen collection locations indicated. Map produced using Natural Earth base layer (https://www.naturalearthdata.com/downloads/50m-cultural-vectors/50m-admin-0-countries-2/) (B) Phenotypes used in the GWA. (C–F): Principal component analysis (PCA) showing the genetic distance between the sampled Melianea marsaeus individuals using a dataset consisting of 205,438 LD pruned biallelic SNPs (C–E) or 1,686 no LD pruned SNPs around the Optix region (F). The scatter plots correspond to the first two principal components (PCs). Points are colored by subspecies (C), geography (D), hindwing black phenotype (E, F). The wing images highlight the color-coded phenotypes. The underlying data can be found at https://zenodo.org/records/19135682. (TIFF) [file pbio.3003742.s019.tiff]

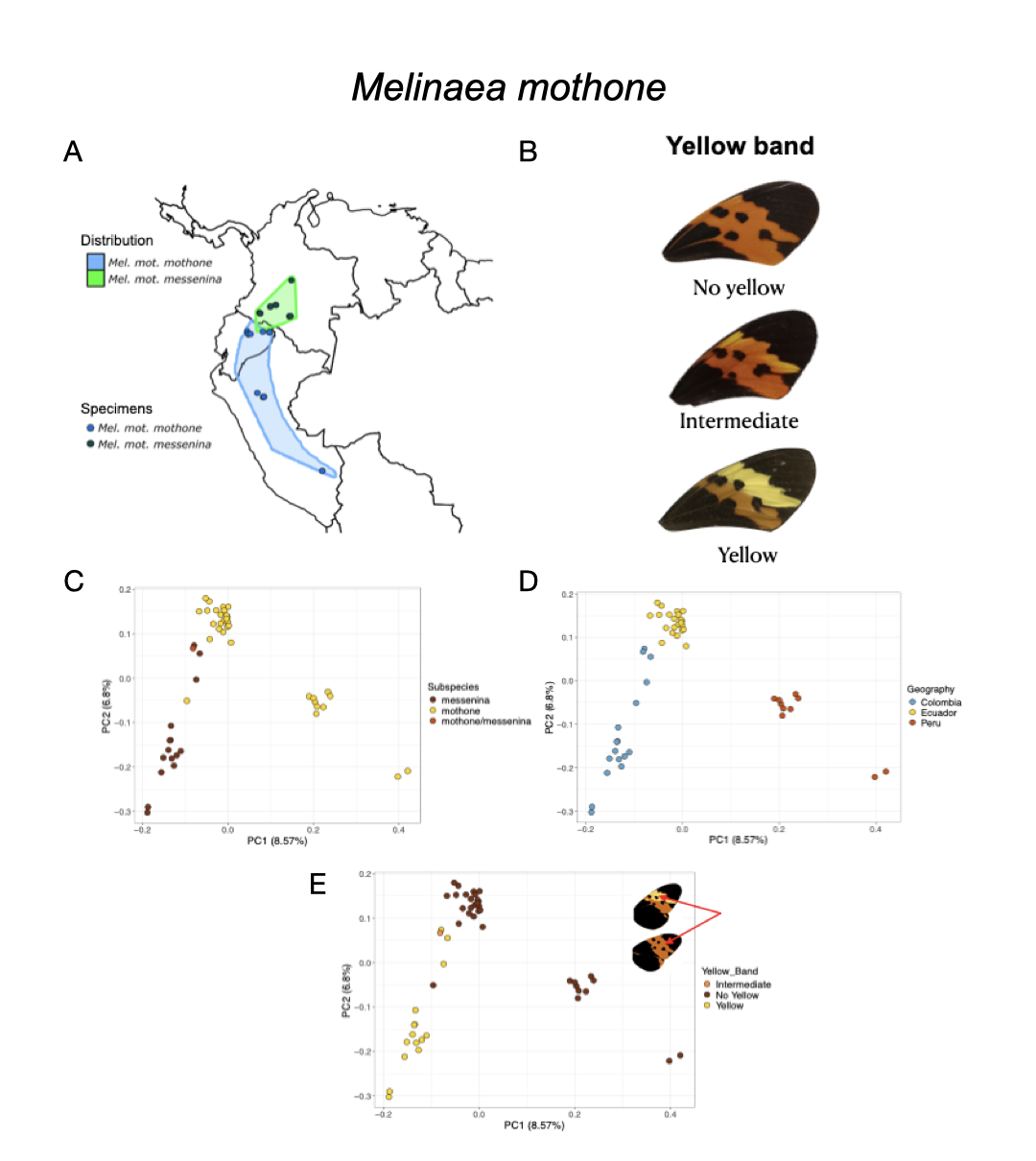

Supplement: S20 Fig — (A) Geographical distribution of Melinaea mpothone subspecies, with specimen collection locations indicated. Map produced using Natural Earth base layer (https://www.naturalearthdata.com/downloads/50m-cultural-vectors/50m-admin-0-countries-2/ (B) Phenotypes used in the GWA. (C–E): Principal component analysis (PCA) showing the genetic distance between the sampled Melinaea mothone individuals using a dataset consisting of 13,652 LD pruned biallelic SNPs. The scatter plots correspond to the first two principal components (PCs). Points are colored by subspecies (C), geography (D), and yellow band phenotype (E). The wing images highlight the color-coded phenotypes. The underlying data can be found at https://zenodo.org/records/19135682. (TIFF) [file pbio.3003742.s020.tiff]

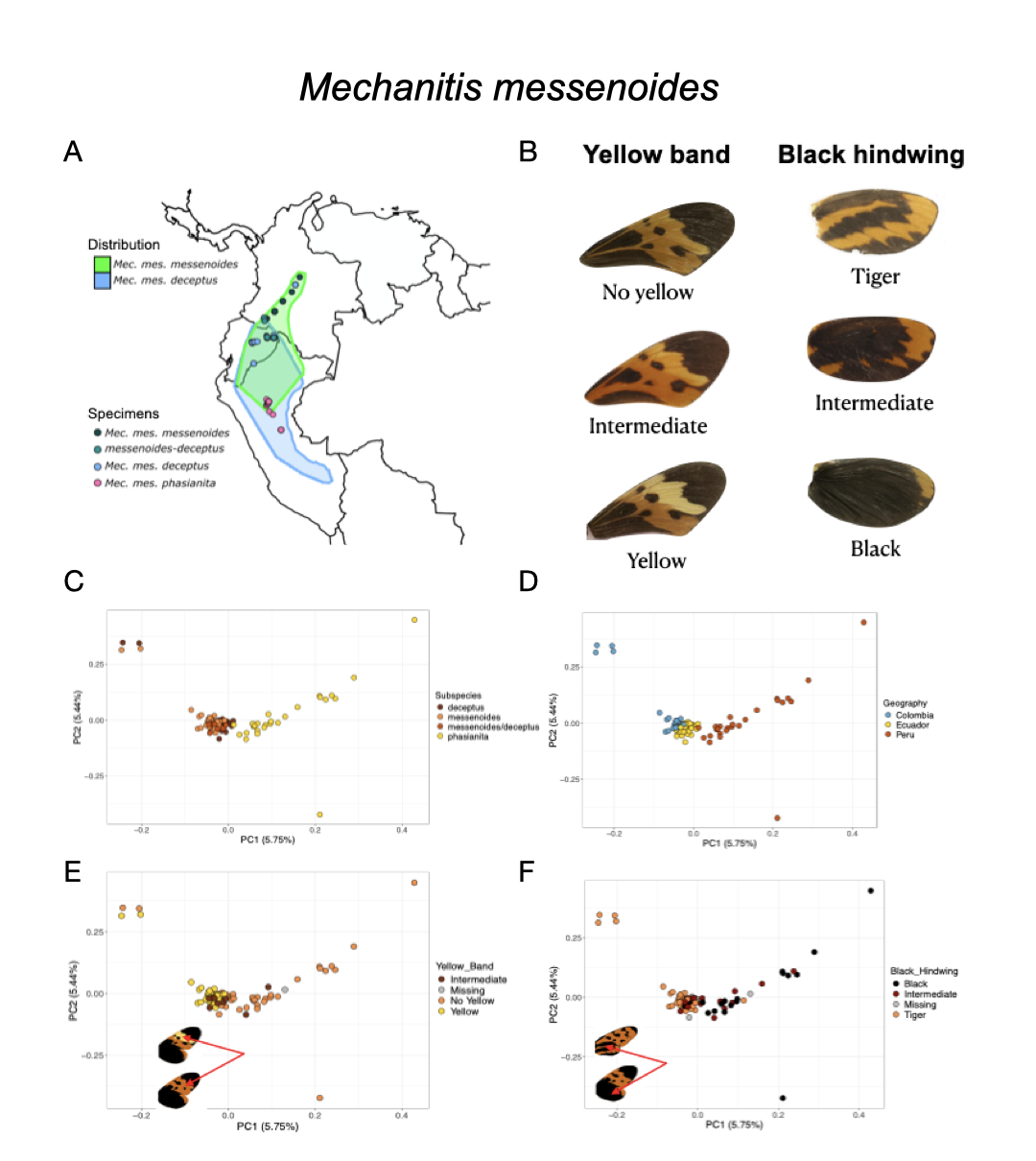

Supplement: S21 Fig — (A) Geographical distribution of Mechanitis messenoides subspecies, with specimen collection locations indicated. Map produced using Natural Earth base layer (https://www.naturalearthdata.com/downloads/50m-cultural-vectors/50m-admin-0-countries-2/ (B) Phenotypes used in the GWA. (C–F): Principal component analysis (PCA) showing the genetic distance between the sampled Mechanitis messenoides individuals using a dataset consisting of 86,312 LD pruned biallelic SNPS. The scatter plots correspond to the first two principal components (PCs). Points are colored by subspecies (C), geography (D), yellow band phenotype (E), and hindwing black phenotype (F). The wing images highlight the color-coded phenotypes. The underlying data can be found at https://zenodo.org/records/19135682. (TIFF) [file pbio.3003742.s021.tiff]

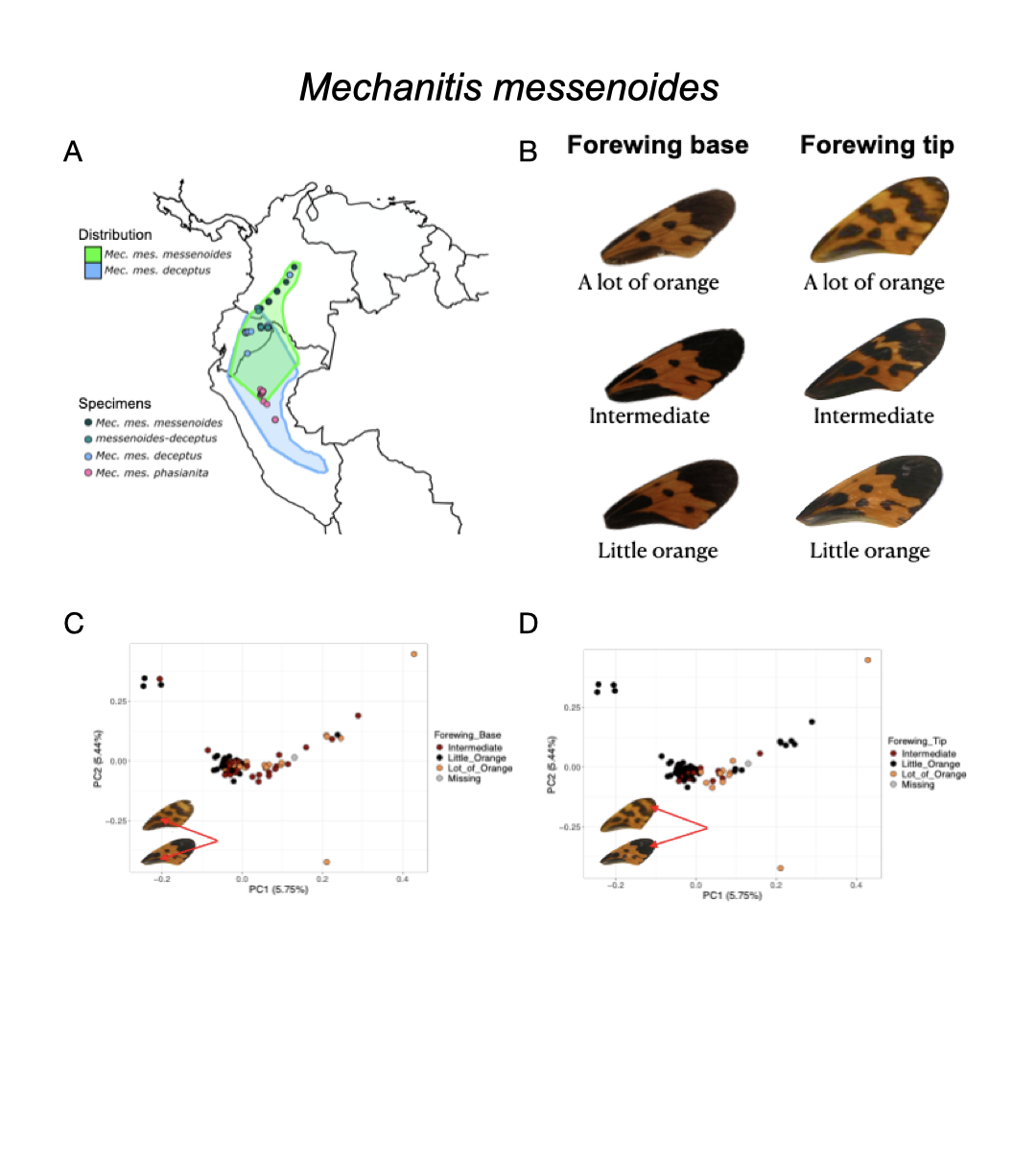

Supplement: S22 Fig — (A) Geographical distribution of Mechanitis messenoides subspecies, with specimen collection locations indicated. Map produced using Natural Earth base layer (https://www.naturalearthdata.com/downloads/50m-cultural-vectors/50m-admin-0-countries-2/ (B) Phenotypes used in the GWA. (C–D): Principal component analysis (PCA) showing the genetic distance between the sampled Mechanitis messenoides individuals using a dataset consisting of 86,312 LD pruned biallelic SNPS. The scatter plots correspond to the first two principal components (PCs). Points are colored by forewing base phenotype (C) and forewing tip phenotype (D). The wing images highlight the color-coded phenotypes. The underlying data can be found at https://zenodo.org/records/19135682. (TIFF) [file pbio.3003742.s022.tiff]

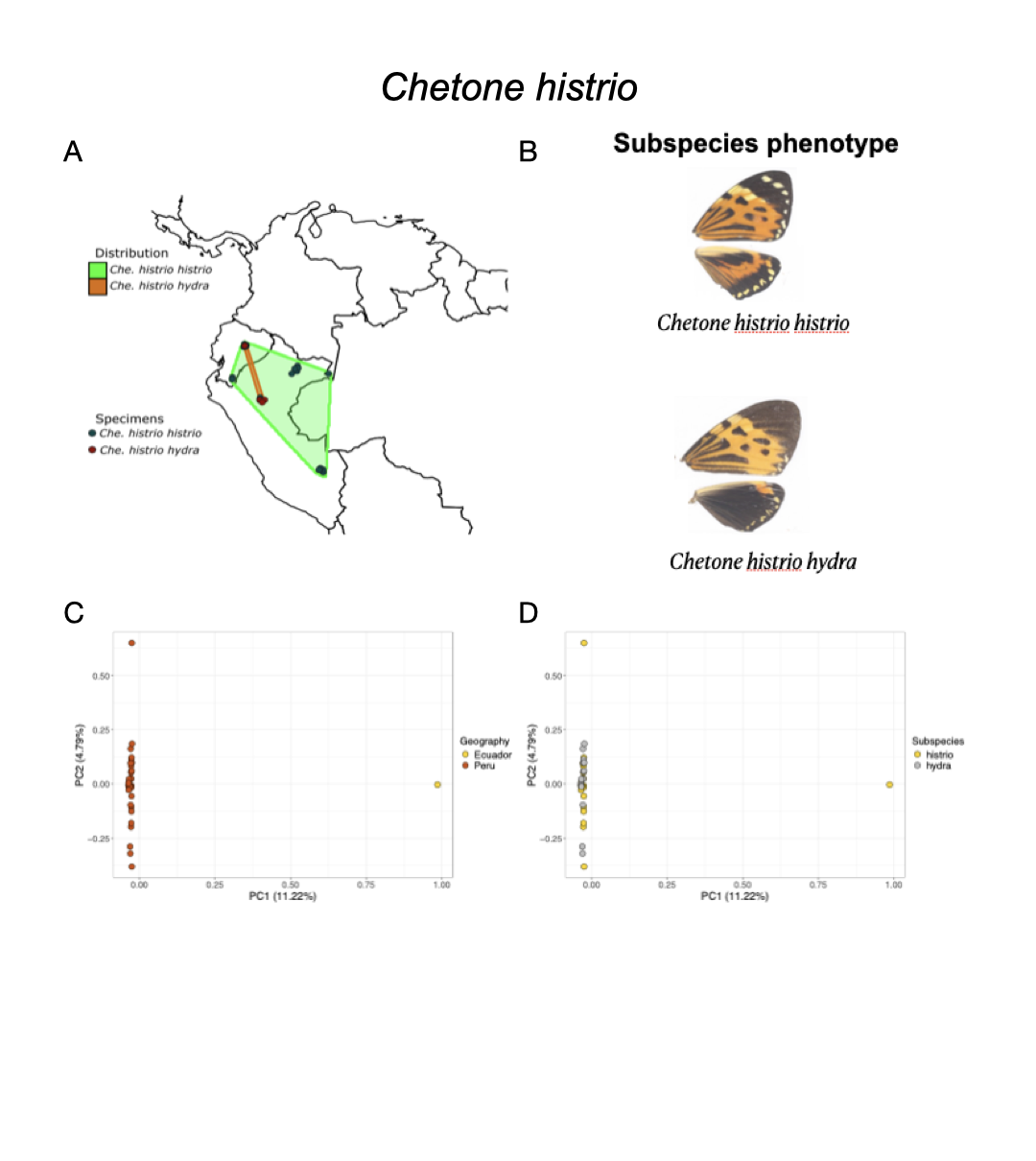

Supplement: S23 Fig — (A) Geographical distribution of Chetone histrio subspecies, with specimen collection locations indicated. Map produced using Natural Earth base layer (https://www.naturalearthdata.com/downloads/50m-cultural-vectors/50m-admin-0-countries-2/ (B) Phenotypes used in the GWA. Principal Component Analysis (PCA) of 1,159,948 LD pruned biallelic SNPs in Chetone histrio with points colored by (C) geography and (D) subspecies. The underlying data can be found at https://zenodo.org/records/19135682. (TIFF) [file pbio.3003742.s023.tiff]

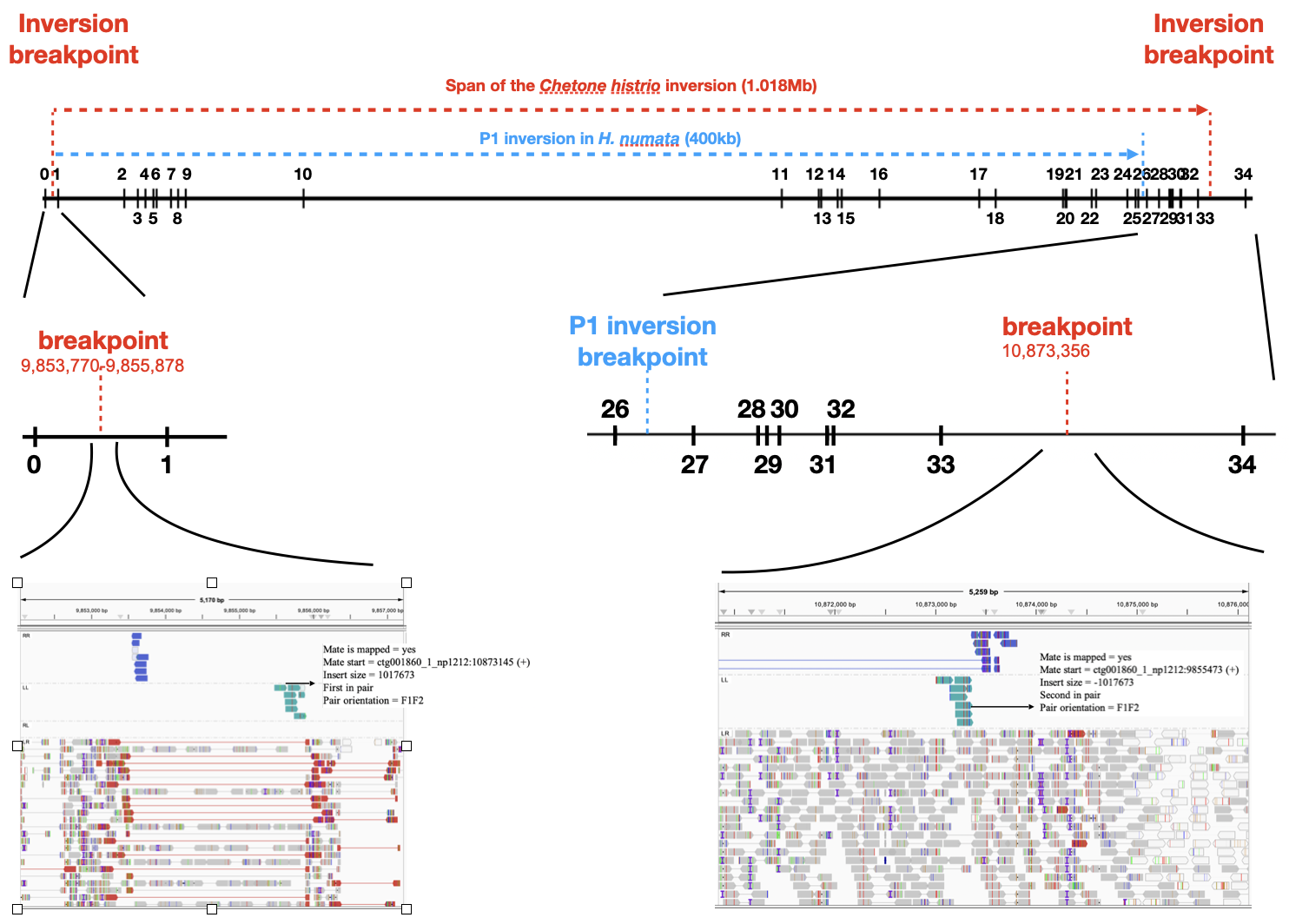

Supplement: S24 Fig — Examination of Illumina read-pair orientation and mapped insert sizes using IGV allows localization of the inversion breakpoints in Chetone histrio. The IGV screenshots are for individual NR15−519, which is heterozygous for the inversion. a) Comparison of the locations of the Chetone histrio and Heliconius numata P1 inversion based on the locations of genes labeled 0−34. b) The left breakpoint in Chetone histrio is located between genes 0 and 1 at ctg001860:9,853,770−9,855,878. A region of poor mapping prevents more accurate inference of the breakpoint. c) The right breakpoint in Chetone histrio is located between genes 33 and 34 at ctg001860:10,873,356. Gene 0–34 refer to: 0: beta-fructofuranosidase; 1: glutaminyl-peptidecyclotransferase; 2: HMEL000021; 3: enoyl-CoA hydratase; 4: Cancer-related nucleoside-triphosphatase; 5: Sur-8/LRR; 6: HMEL032678; 7: HMEL002023g1; 8: HMEL002023g2; 9: HMEL002024; 10: cortex; 11: parn; 12: HMEL000027; 13: ARP-like; 14: ATP synthase subunit f; 15: proteasome 26S non ATPasesubunit 4; 16: zinc phosphodiesterase; 17: serine/threonine-proteinkinase; 18: WD repeat-containing protein 19; 19: HMEL013472; 20: WAS protein family homologue 1; 21: Domeless; 22: HMEL032681; 23: HMEL032683; 24: mitogen-activated protein kinase; 25: DNA excision repair protein ERCC-6; 26: penguin; 27: thymidylate kinase; 28: caspase-activated DNase; 29: ribosome biogenesis regulatory protein; 30: INO80 complex subunit C; 31: uncharacterized WD repeat-containing protein C2E1P5.05; 32: Sr protein; 33: HMEL000048; 34: HMEL000049. (TIFF) [file pbio.3003742.s024.tiff]

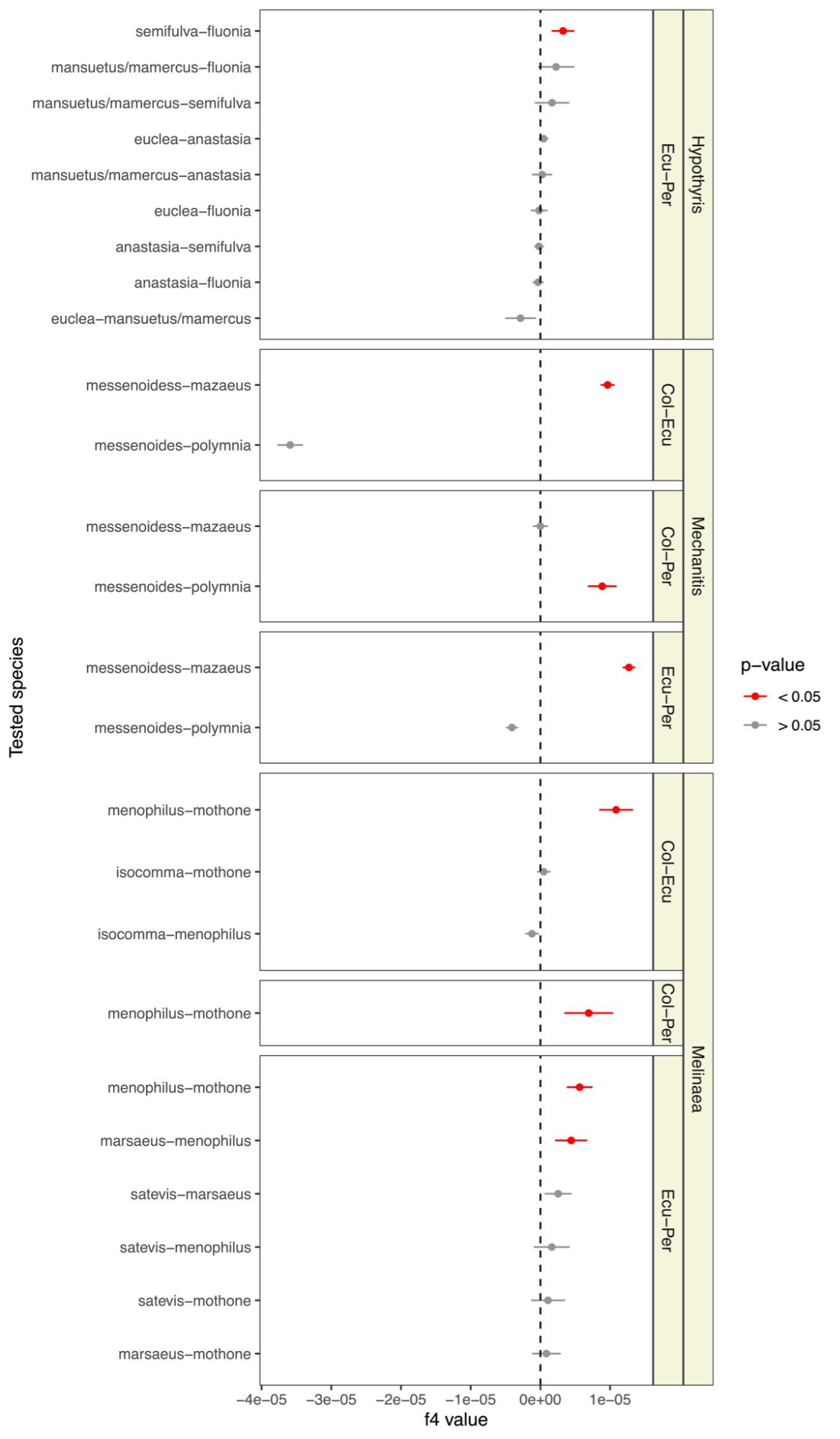

Supplement: S25 Fig — Comparisons are shown across different geographic regions (Colombia-Ecuador, Colombia-Peru, and Ecuador-Peru). Each point represents an f4 value, with standard errors. Red points are significantly different from zero (p < 0.05). Significantly positive f4 value suggests excess allele sharing between the tested species, consistent with interspecific gene flow. Details of the taxa tested are shown in S5 Table. The underlying data can be found at https://zenodo.org/records/19135682. (TIFF) [file pbio.3003742.s025.tiff]

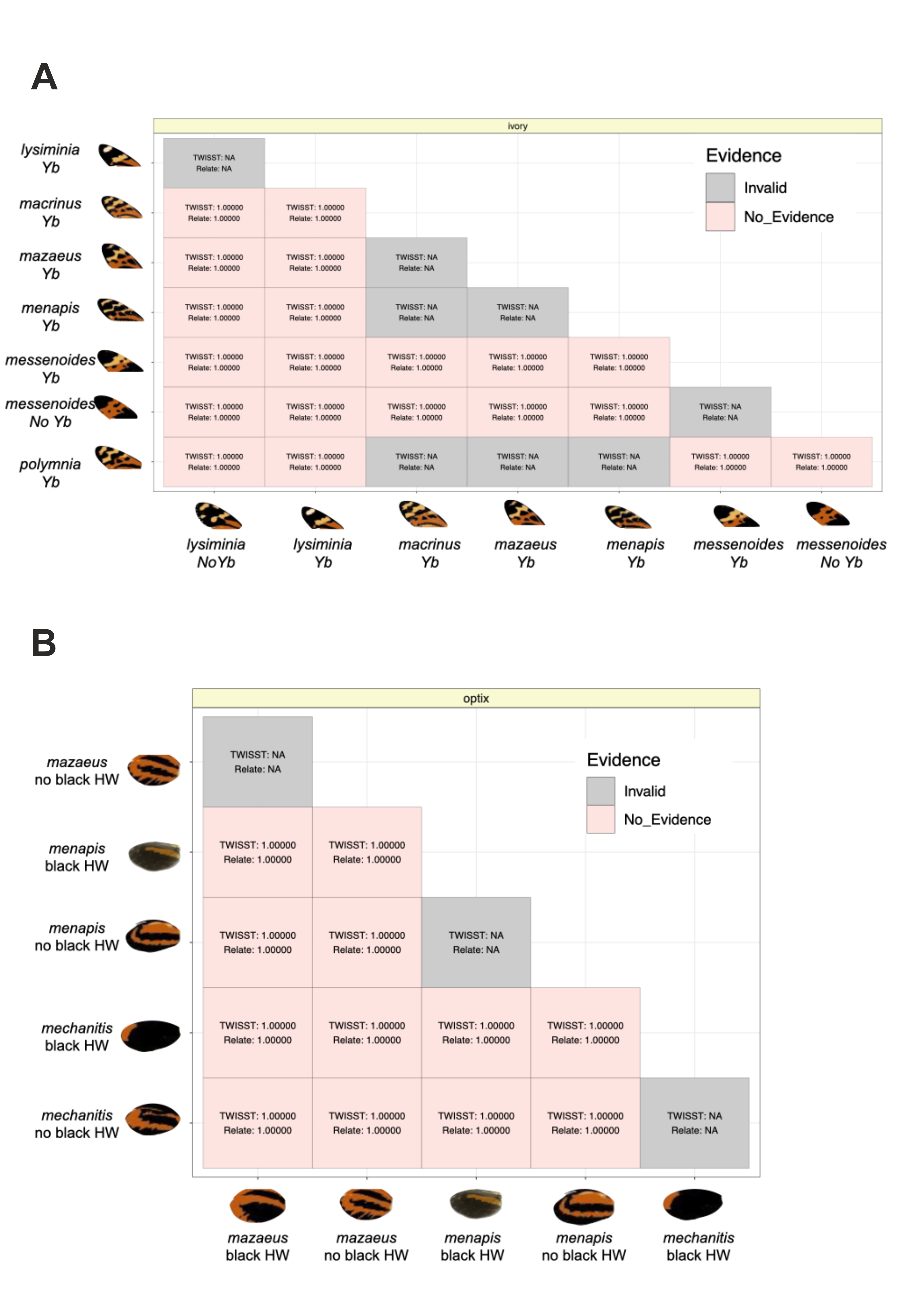

Supplement: S26 Fig — Comparisons are shown between species with different forewing yellow band (top) and hindwing black vs orange (bottom) phenotypes. Each cell in the matrix represents a comparison between a pair of species, with rows and columns labeled by species and wing phenotype. No evidence of introgression (pink) is found in any comparison. The Twisst and Relate p-values, based on a block permutation test, are displayed in each cell. ”NA“ indicates intraspecific or invalid comparisons (gray). P-values of 1.00000 indicate that no introgression-compatible topologies were observed within the GWAS peak region, making it impossible to compute a p-value. For Relate, “low” indicates that the analyses included fewer than 20 samples and could not be run. Species and wing phenotypes are depicted along the matrix’s margins with corresponding wing photographs. Details of the taxa tested are shown in S4 Table. The underlying data can be found at https://zenodo.org/records/19135682. (TIF) [file pbio.3003742.s026.tif]

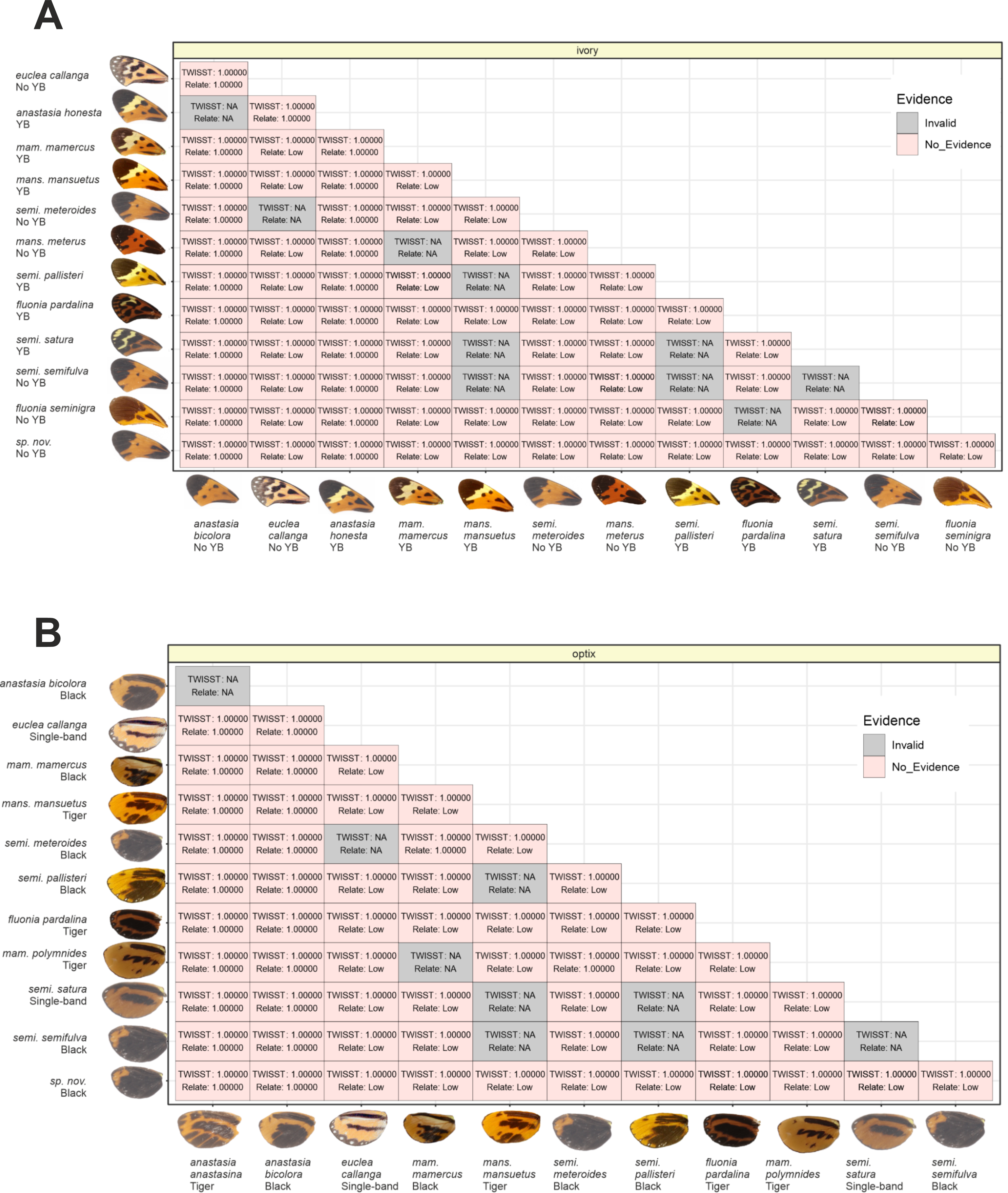

Supplement: S27 Fig — Comparisons are shown between species with different forewing yellow band (top) and hindwing black vs orange (bottom) phenotypes. Each cell in the matrix represents a comparison between a pair of species, with rows and columns labeled by species and wing phenotype. No evidence of introgression (pink) is found in any comparison. The Twisst and Relate p-values, based on a block permutation test, are displayed in each cell. ”NA“ indicates intraspecific or invalid comparisons (gray). P-values of 1.00000 indicate that no introgression-compatible topologies were observed within the GWAS peak region, making it impossible to compute a p-value. For Relate, “low” indicates that the analyses included fewer than 20 samples and could not be run. Species and wing phenotypes are depicted along the matrix’s margins with corresponding wing photographs. Details of the taxa tested are shown in S4 Table. The underlying data can be found at https://zenodo.org/records/19135682. (TIF) [file pbio.3003742.s027.tif]

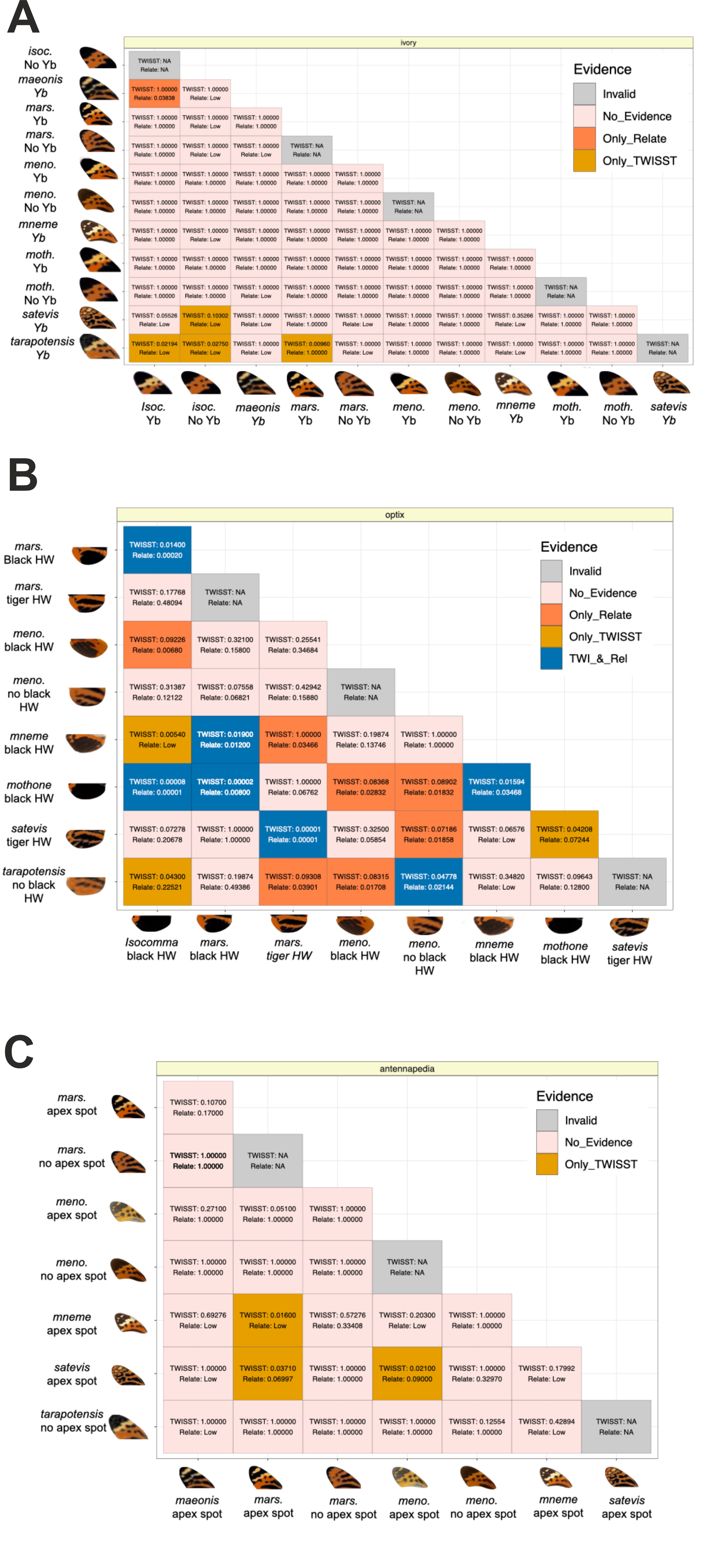

Supplement: S28 Fig — Comparisons are shown between species with different forewing yellow band (top), hindwing black vs orange (middle), and forewing apex spot (bottom) phenotypes. Each cell in the matrix represents a comparison between a pair of species, with rows and columns labeled by species and wing phenotype. The matrix is color-coded to indicate the type of evidence detected: no evidence of introgression (pink), evidence from Relate only (orange), evidence from Twisst only (ochre), or evidence from both Relate and Twisst (blue). The Twisst and Relate p-values, based on a block permutation test, are displayed in each cell. “NA“ indicates intraspecific or invalid comparisons (gray). P-values of 1.00000 indicate that no introgression-compatible topologies were observed within the GWAS peak region, making it impossible to compute a p-value. For Relate, “low” indicates that the analyses included fewer than 20 samples and could not be run. Species and wing phenotypes are depicted along the matrix’s margins with corresponding butterfly illustrations. Abbreviations for species names are as follows: meno.: menophius; mars.: marsaeus; isoc.: isocomma; moth.: mothone. Details of the taxa tested are shown in S4 Table. The underlying data can be found at https://zenodo.org/records/19135682. (TIF) [file pbio.3003742.s028.tif]

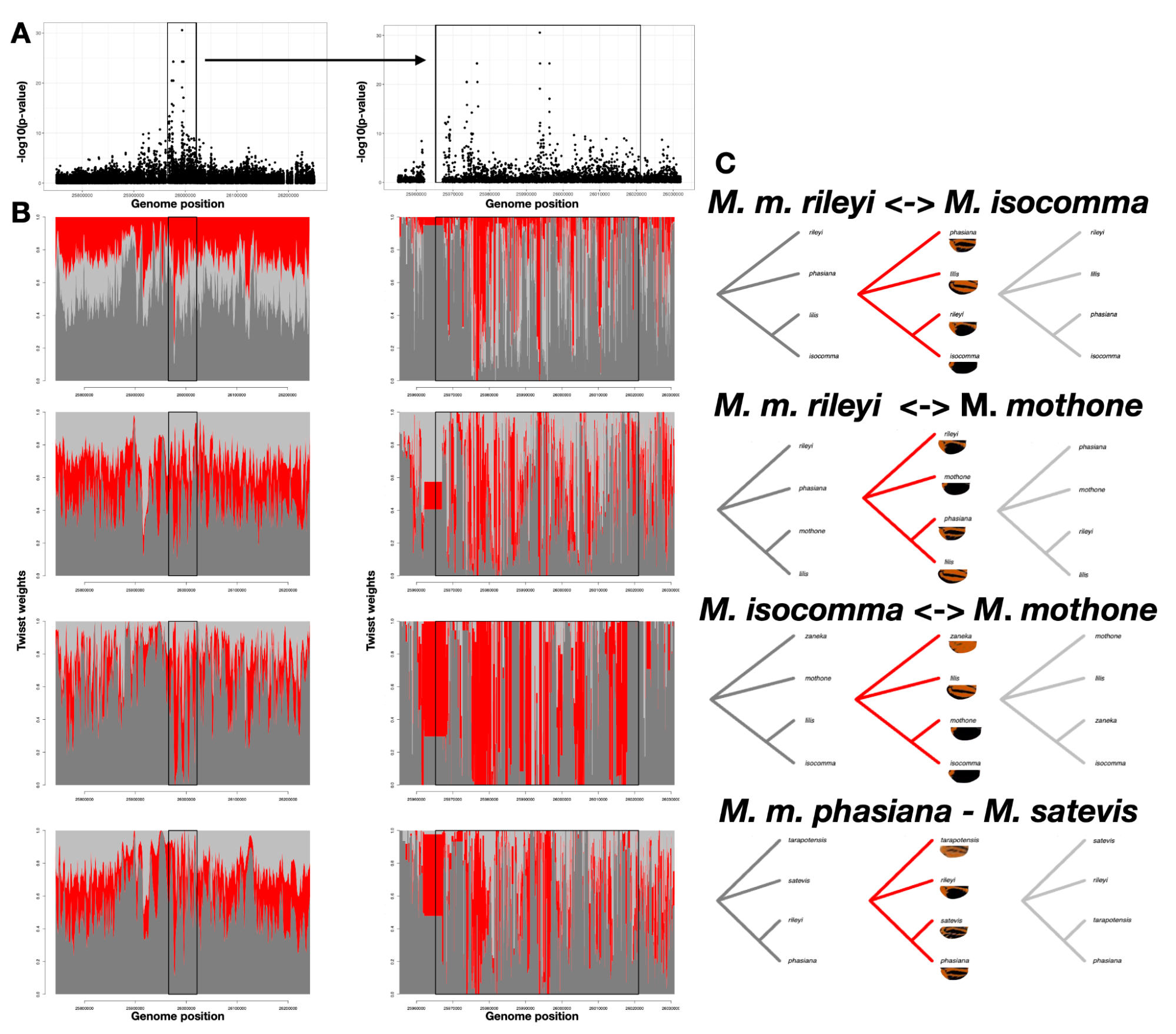

Supplement: S29 Fig — (A) Melinaea marsaeus GWA Manhattan plot near optix. The black rectangle highlights the strongest association peak, which is shown zoomed in on the right panel. (B) Twisst weight bar plots displaying local phylogenetic topology support across the highlighted region in a. Each row corresponds to a different species pair, with the y-axis indicating the Twisst weight, which quantifies how congruent the local genealogy is with one of the three topologies shown in (C) The left bar plots provide a broader genomic context and include a smoothing function to reduce noise, while the right bar plots shows a zoom in of the region without smoothing. Higher red values indicate greater support for an introgressed topology, while the two shades of gray correspond to the two alternative topologies expected for unrooted trees based on four taxa. (C) Most likely marginal coalescent trees for each species pair tested, inferred using RELATE. The underlying data can be found at https://zenodo.org/records/19135682. (TIFF) [file pbio.3003742.s029.tiff]

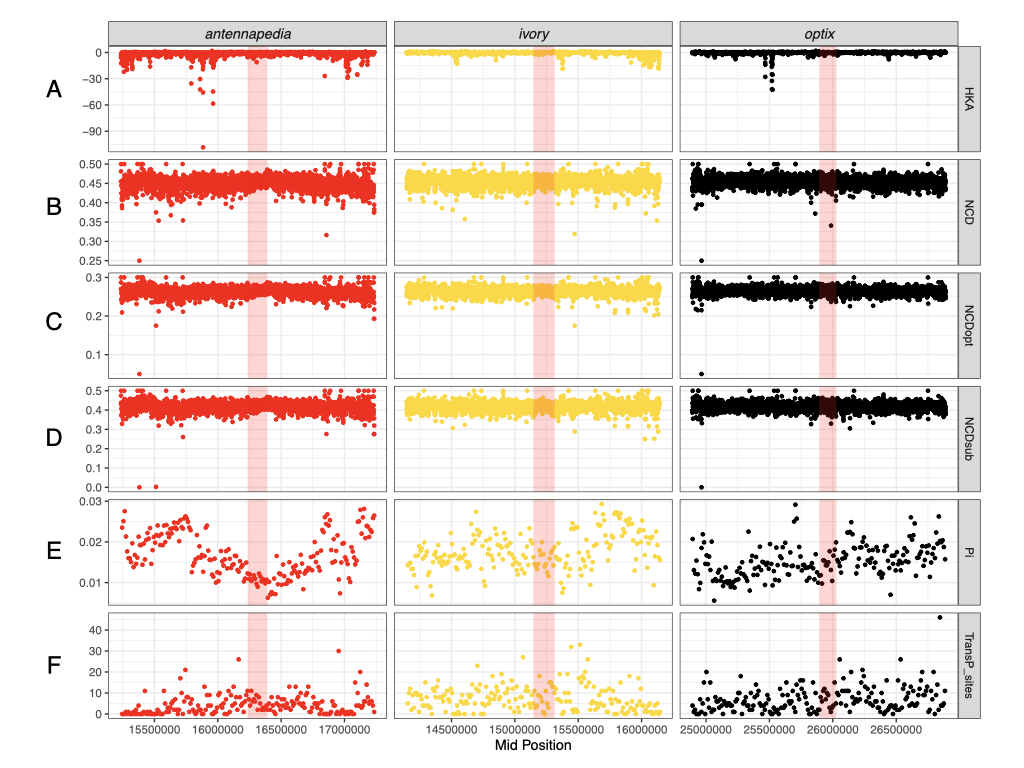

Supplement: S30 Fig — These analyses included Melinaea isocomma, Melinaea mothone, Melinaea marsaeus, Melinaea menophilus, Melinaea satevis, and Melinaea tarapotensis. The red-shaded regions indicate the location of the GWA peaks in the genes antennapedia, ivory and optix. (A) HKAtrans statistics computed in 1 kb windows with a 500 bp sliding step. Melinaea ludovica was used to determine the ancestral state. Positive HKAtrans values indicate an excess of shared polymorphisms relative to divergence, which is consistent with ancient trans-species balancing selection. (B) NCD2trans statistics computed in 1 kb windows with a 500 bp sliding step. This statistic measures allele frequency deviations from neutral expectations across multiple species. Values closer to 0 suggest ancient trans-species balancing selection. (C) NCD2trans-opt statistics computed in 1 kb windows with a 500 bp sliding step. This statistic is a variant of NCD2trans that optimizes the target frequency at neutrality. Values closer to 0 suggest ancient trans-species balancing selection. (D) NCD2trans-sub statistics computed in 1 kb windows with a 500 bp sliding step. A variant of NCD2trans that treats substitutions and polymorphisms separately. Values closer to 0 suggest ancient trans-species balancing selection. (E) Multispecies nucleotide diversity (π) computed in 10 kb windows. Larger values are indicative of ancient trans-species balancing selection. (F) Number of transpolymorphic sites computed in 10 kb windows. Larger values are indicative of ancient trans-species balancing selection. The underlying data can be found at https://zenodo.org/records/19135682. (TIFF) [file pbio.3003742.s030.tiff]

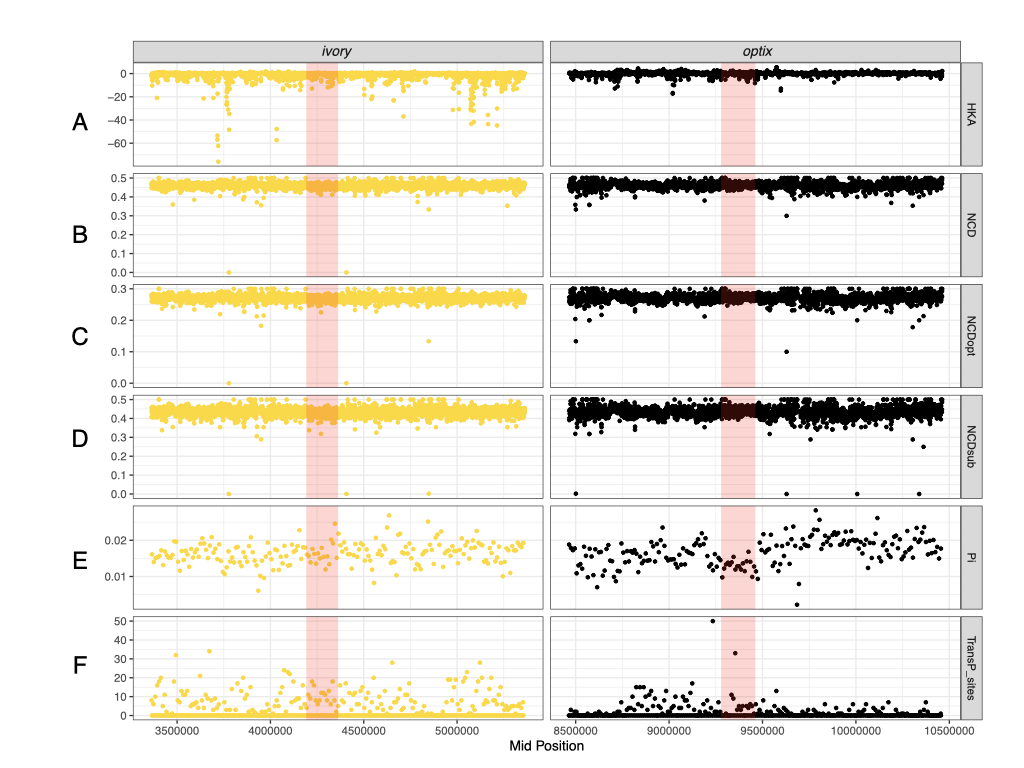

Supplement: S31 Fig — These analyses included Hypothyris anastasia, Hypothyris euclea, Hypothyris ninonia, and Hypothyris semifulva. The red-shaded regions indicate the location of the GWA peaks in the genes ivory and optix. (A) HKAtrans statistics computed in 1 kb windows with a 500 bp sliding step. Hyalyris antea was used to determine the ancestral state. Positive HKAtrans values indicate an excess of shared polymorphisms relative to divergence, which is consistent with ancient trans-species balancing selection. (B) NCD2trans statistics computed in 1 kb windows with a 500 bp sliding step. This statistic measures allele frequency deviations from neutral expectations across multiple species. Values closer to 0 suggest ancient trans-species balancing selection. (C) NCD2trans-opt statistics computed in 1 kb windows with a 500 bp sliding step. This statistic is a variant of NCD2trans that optimizes the target frequency at neutrality. Values closer to 0 suggest ancient trans-species balancing selection. (D) NCD2trans-sub statistics computed in 1 kb windows with a 500 bp sliding step. A variant of NCD2trans that treats substitutions and polymorphisms separately. Values closer to 0 suggest ancient trans-species balancing selection. (E) Multispecies nucleotide diversity (π) computed in 10 kb windows. Larger values are indicative of ancient trans-species balancing selection. (F) Number of transpolymorphic sites computed in 10 kb windows. Larger values are indicative of ancient trans-species balancing selection. The underlying data can be found at https://zenodo.org/records/19135682. (TIFF) [file pbio.3003742.s031.tiff]

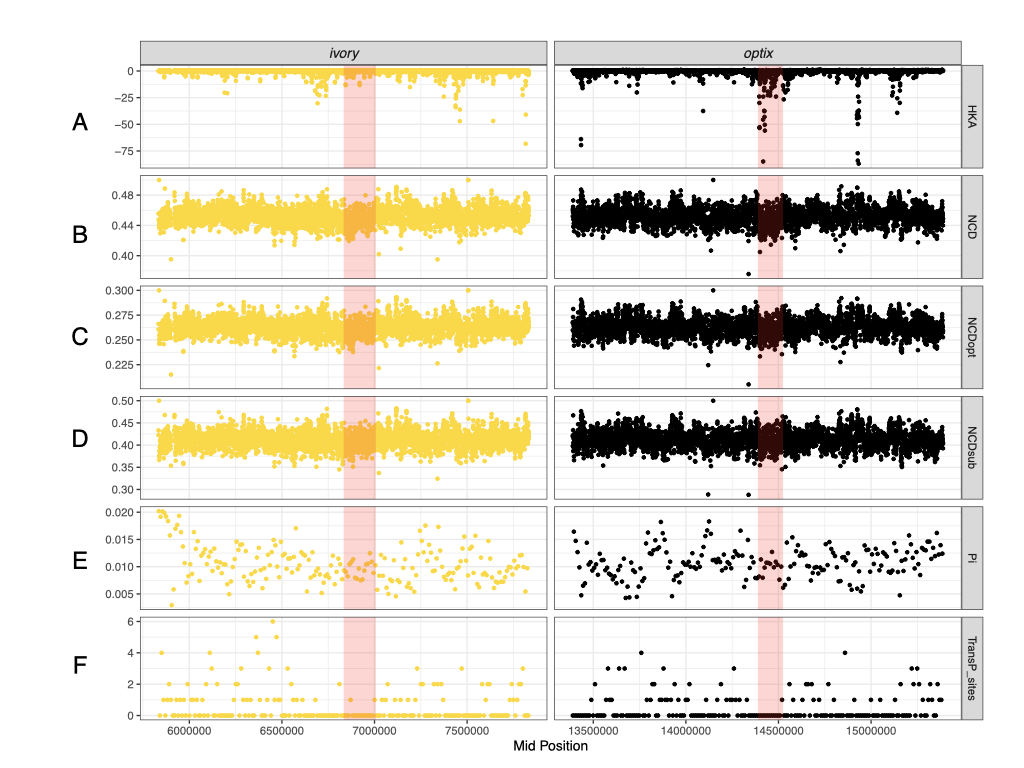

Supplement: S32 Fig — These analyses included Hypothyris anastasia, Hypothyris euclea, Hypothyris ninonia, and Hypothyris semifulva. The red-shaded regions indicate the location of the GWA peaks in the genes ivory and optix. (A) HKAtrans statistics computed in 1 kb windows with a 500 bp sliding step. Hyalyris antea was used to determine the ancestral state. Positive HKAtrans values indicate an excess of shared polymorphisms relative to divergence, which is consistent with ancient trans-species balancing selection. (B) NCD2trans statistics computed in 1 kb windows with a 500 bp sliding step. This statistic measures allele frequency deviations from neutral expectations across multiple species. Values closer to 0 suggest ancient trans-species balancing selection. (C) NCD2trans-opt statistics computed in 1 kb windows with a 500 bp sliding step. This statistic is a variant of NCD2trans that optimizes the target frequency at neutrality. Values closer to 0 suggest ancient trans-species balancing selection. (D) NCD2trans-sub statistics computed in 1 kb windows with a 500 bp sliding step. A variant of NCD2trans that treats substitutions and polymorphisms separately. Values closer to 0 suggest ancient trans-species balancing selection. (E) Multispecies nucleotide diversity (π) computed in 10 kb windows. Larger values are indicative of ancient trans-species balancing selection. (F) Number of transpolymorphic sites computed in 10 kb windows. Larger values are indicative of ancient trans-species balancing selection. The underlying data can be found at https://zenodo.org/records/19135682. (TIFF) [file pbio.3003742.s032.tiff]

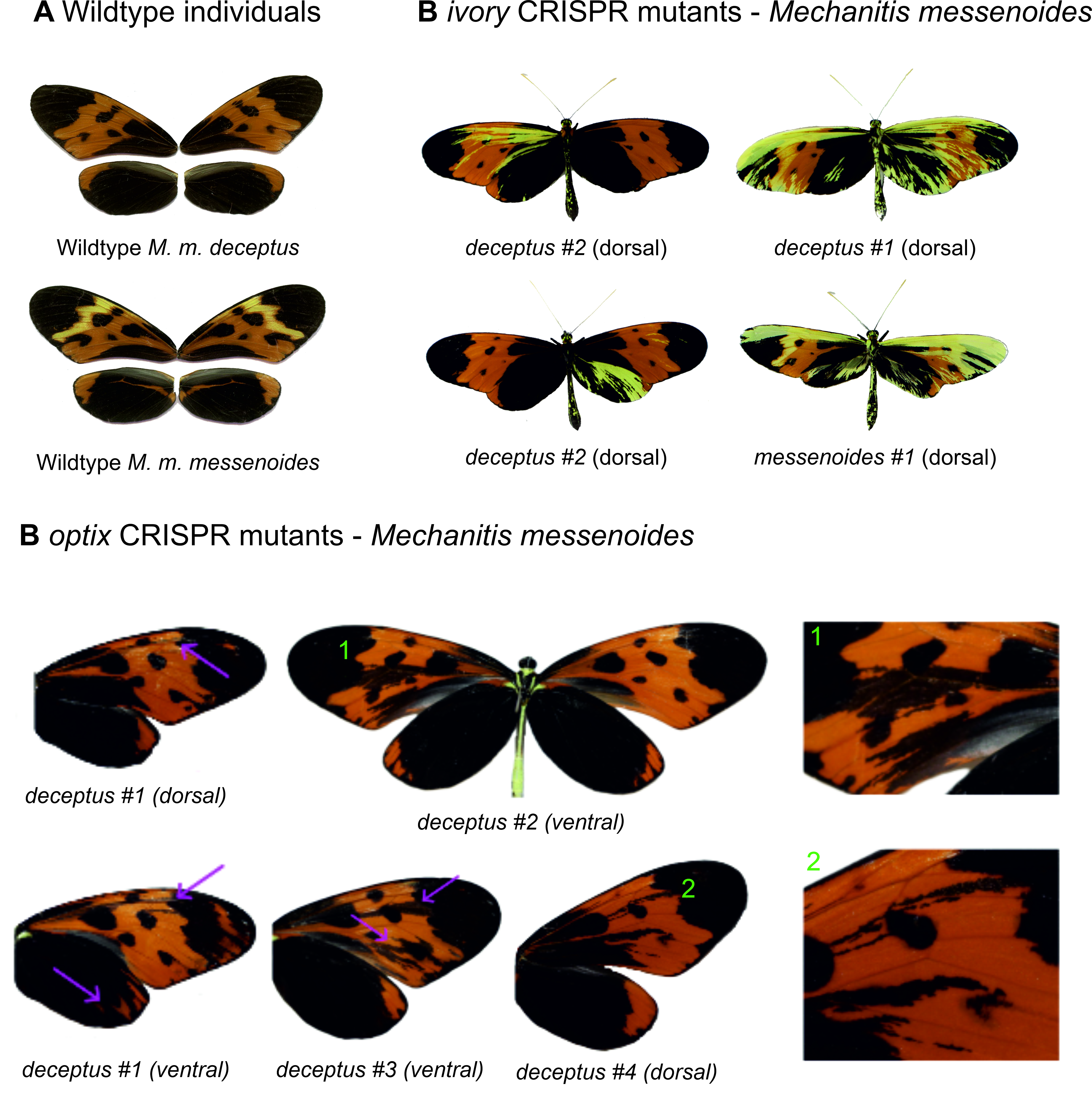

Supplement: S33 Fig — Mutant phenotypes can be recognized through their asymmetry (wildtype individuals have symmetric left and right wings. (A) Wildtype individuals of the messenoides and deceptus subspecies. (B) ivory mutants in which orange and black scales have turned yellow. (C) optix mutants in which orange scales have turned black. (TIF) [file pbio.3003742.s033.tif]

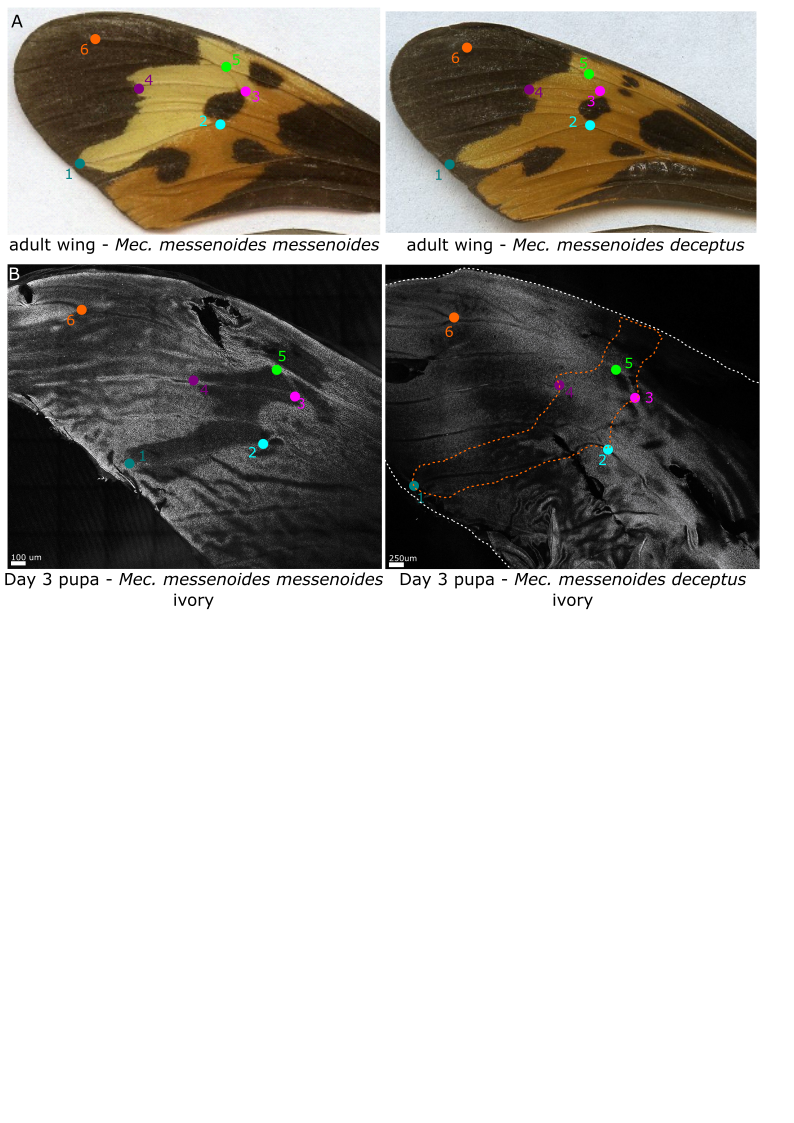

Supplement: S34 Fig — Comparing nonyellow-banded Mec. messenoides deceptus and yellow banded Mec. messenoides messenoides shows absence of ivory RNA in the yellow band region of the forewing. The colored dots indicate vein-based wing landmarks, demarcating the yellow band region which lacks ivory expression in Mec. messenoides messenoides. In the nonyellow-banded Mec. messenoides deceptus, the red dotted line demarcates the approximate region corresponding to the yellow band region. (TIFF) [file pbio.3003742.s034.tiff]

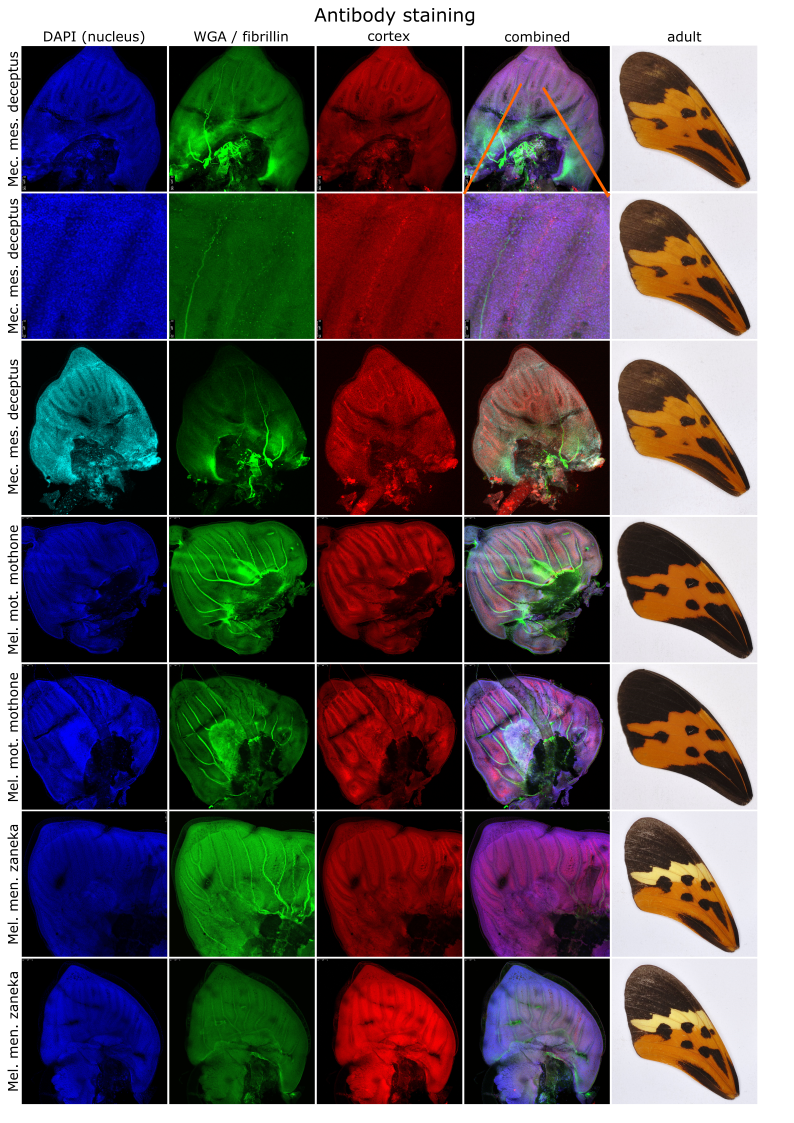

Supplement: S35 Fig — Cortex is expressed across the entire forewings of early fifth instar larvae. Each row shows a set of photos taken on a confocal microscope, with each column showing a different channel (column 1: nuclear DAPI staining (405 nm), column 2: wheat germ agglutinin (WGA) that stains the nuclear membrane (488 nm) or fibrillin, which stains the nucleolus: column 3: Cortex antibody (555 nm); column 4: an overlay image with all three channels combined). The last column shows the adult phenotype. Row 1–3: Mechanitis messenoides deceptus, with row 2 being a more zoomed image of row 1. Row 4–5: Melinaea mothone mothone (no yellow band). Row 6–7: Melinaea menophilus zaneka (with yellow band). (TIFF) [file pbio.3003742.s035.tiff]

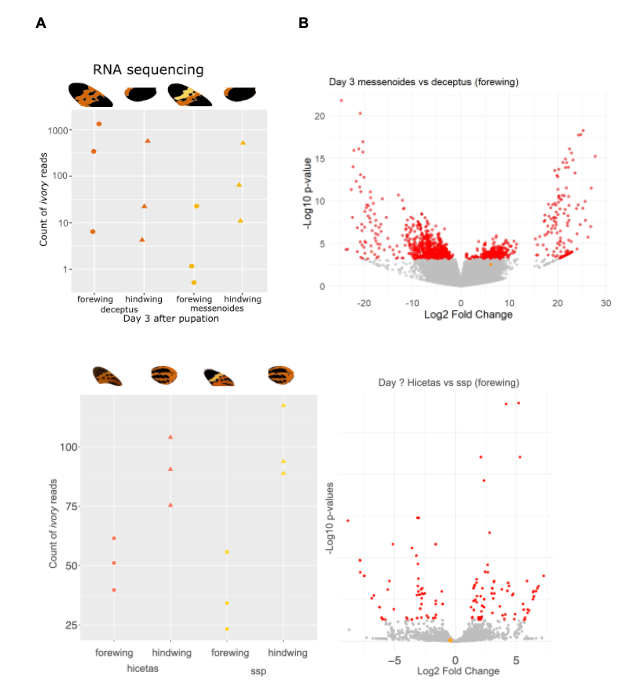

Supplement: S36 Fig — (A) Normalized ivory expression levels in the forewing and hindwing across subspecies of Mechanitis messenoides (top) and Melinaea menophilus (bottom). (B) Volcano plots of genome-wide differential expression analysis comparing yellow-banded versus nonyellow-banded forewings in Mechanitis messenoides (top) and Melinaea menophilus (bottom). Red dots indicate significantly differentially expressed genes; the orange dot corresponds to ivory. The underlying data can be found at https://zenodo.org/records/19135682. (TIFF) [file pbio.3003742.s036.tiff]

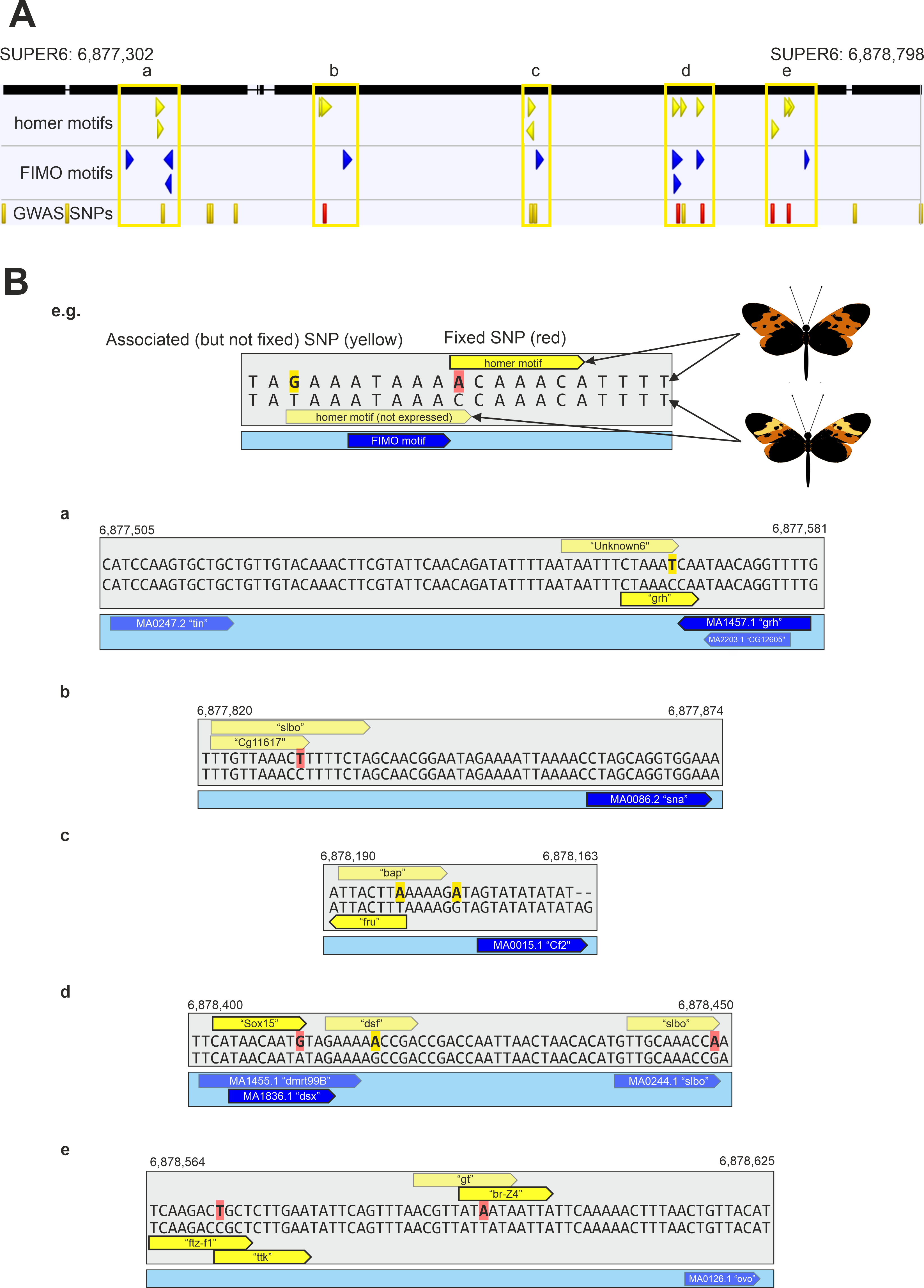

Supplement: S37 Fig — (A) The GWAS peak region ranging to one (weakly- or non-associated) SNP either side of the fixed and most highly associated SNPs (SUPER_6:6877302–6878798) annotated with the motifs identified by homer (yellow arrows) which appear in 100% of sequences for one form and less than 5% of sequences for the other. Highly variable or repetitive motifs which occurred less than 3 times per sequence were excluded. Motifs identified by FIMO110 which fall within 50 bp of an associated SNP detected by GWAS are shown in blue. Fixed SNPs are highlighted in red, whilst associated SNPs which are not completely fixed are shown in yellow. (B) Regions surrounding the homer motifs and closest FIMO motifs are shown in detail. The nucleotide sequences are the consensus sequence from the nonyellow-banded form (deceptus, top) and yellow-banded reference form (messenoides, bottom). Homer109 motifs which are enriched in the nonyellow-banded sequences are shown above this consensus sequence, whilst motifs enriched in the yellow-banded form are shown below. FIMO motifs were detected in both sets of sequences. Faded motif labels indicate transcription factors that are not expressed in forewing pupal wing discs. Associated/fixed SNPs are highlighted in the nonyellow-banded sequence according to the nucleotide present. The names of the FIMO motifs correspond to the JASPAR database, whilst the names of the homer motifs come from the “best-guess” matching motif identified by homer. The underlying data can be found at https://zenodo.org/records/19135682. (TIF) [file pbio.3003742.s037.tif]

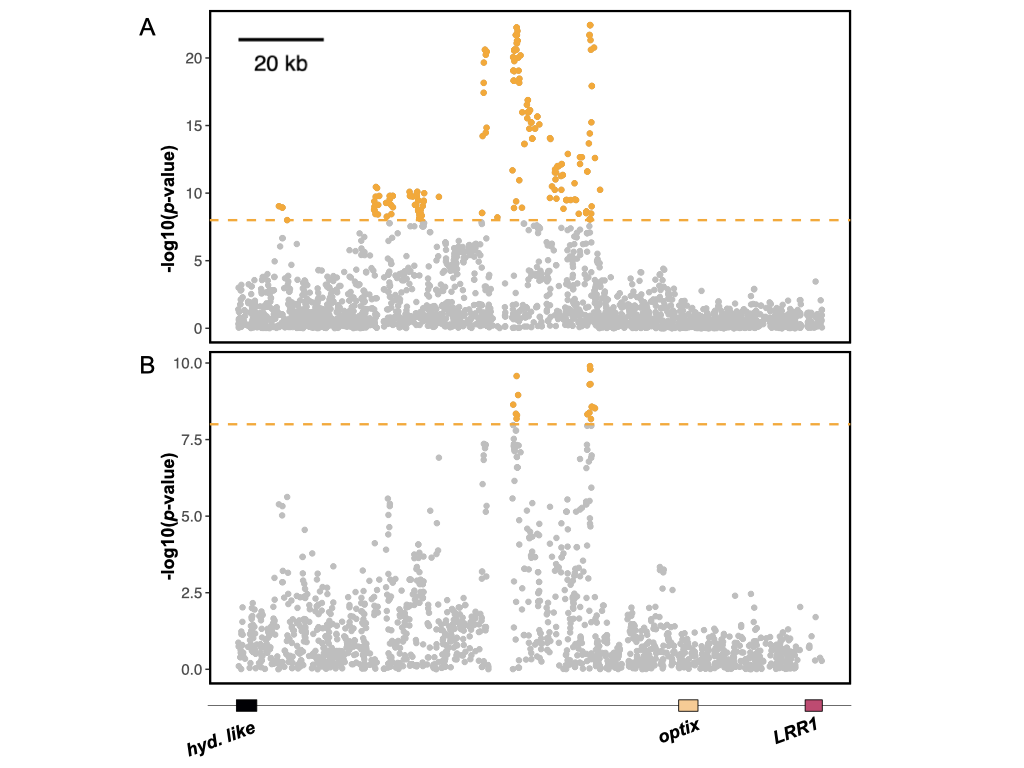

Supplement: S38 Fig — (A) GWA using phenotype values based on manual, categorical grouping. Individuals were classed as either 0 (semi-melanised, “tiger-patterned” hindwing) or 1 (fully melanic, black hindwing). (B) GWA using phenotype values generated through a quantitative, color pattern analysis based approach using Patternize54. SNPs above the Bonferroni-corrected significance threshold (dashed orange line) are colored orange. The underlying data can be found at https://zenodo.org/records/19135682. (TIFF) [file pbio.3003742.s038.tiff]
