# Supplementary material for: Genetic parallelism underpins convergent mimicry coloration in Lepidoptera across 120 million years of evolution
Source: PLoS Biol. 2026 Apr 30;24(4):e3003742. doi: 10.1371/journal.pbio.3003742 (PMC13132254; doi:10.1371/journal.pbio.3003742)
Supplement: Attachment — Submitted filename: Reviewers comments and response.docx [file pbio.3003742.s048.docx]

We thank the three reviewers for reading our manuscript and for their positive comments. Please find below in red our responses to the minor but helpful comments they have made (line numbers refer to the track changes document). In addition to these suggested changes we have made other minor edits to improve clarity. These changes are mostly in the methods section (lines 299-302, 310-311, 345-348, 372-380, 465-467). All changes made can be seen in the track changes version.

We note that the following pieces of information are at the moment missing:

1) Accession numbers for the two of the genome assemblies used (line 374)
2) Accessions for some RNAseq data (Table S1)

These data have been submitted to NCBI and we are waiting for accession numbers. Accessions for all other sequences have been provided in Table S1.

Kanchon Dasmahapatra

Reviewer #1: This is a beautiful study examining the genetic basis of convergent wing color patterns in mimicry rings. The authors use a rigorous combination of genome sequencing, sophisticated genomic analyses, QTL mapping, CRISPR genome editing, and gene expression studies to identify the genetic basis of color polymorphisms in eight species. Remarkably, the authors find that the same two genes (optix and ivory) underlie convergent color patterns in species that have diverged over 120 million years ago. These results contrast with recent meta-analyses, which have shown that the same genes are used in convergent phenotypes when species are closely related, but not in more distantly related species. Furthermore, the authors show that even in closely related species, gene reuse is not due to allele sharing resulting from hybridization. This result is also in contrast to recent studies in other systems. Together, these results suggest that there are genetic constraints that

limit the genes that can be involved in the evolution of color patterns in these mimicry rings. I think this will quickly be a classic paper in the literature on the genetic basis of repeated evolution.

The manuscript is very well-written with clear figures. I just have a few minor points where I found the writing or figures were not completely clear. Also, a note to the authors to please use page and line numbers to facilitate comments!

1. In the second paragraph of the main text, the sentence: "Where genes are reused, convergence may result from independent evolution of the same phenotype…" is unclear. I think you mean "convergence may result from independent mutation at the same gene". Otherwise, you are just defining convergence.

> Agreed. We have now changed the wording to the suggested one (lines 68-69).

2. In the same paragraph, reference 10 is used to make the point that the same phenotypes can evolve from ancestral standing variation. I think that Colosimo et al. 2005 Science would be a better reference, as it specifically is focused on the genetics of repeated phenotypic evolution and the Jones et al. 2012 Nature paper is focused on repeated adaptation.

> We have replaced Jones et al 2012 with Colosimo et al 2005.

3. In the paragraph on the forewing yellow band in Ithomiini butterflies, it would be helpful to provide more information about what the E230 cis-regulatory element is and how it relates to the ivory micro-RNA.

> Brief additional detail has now been added (line 123) .

4. In the first paragraph on hindwing melanisation in Ithomiini butterflies, it is mentioned that additional associated SNPs in some species are likely due to population structure. But, is there a way to deal with this confounding factor in the association mapping analyses?

> For the GWAS analyses we have used GEMMA which uses a kinship matrix among samples to deal with population structure. However, for some of our species, the phenotypes are perfectly correlated with geography; this can be seen in the PCA plots shown in S17-23 Fig. In such cases GEMMA, and other GWAS software, are unable to deal with this confounding factor.

5. In the last sentence of the paragraph on "Genetic architecture of colour patterning in the moth…:, I would move the phrase "between lineages that diverged ~120 MYA" to the very end of the sentence.

> Changed as suggested (line 192-194).

6. In the first paragraph of the section "Repeatable and predictable evolution", I don't really see the evidence in the paper for "modifiers located next to these switches…". Indeed, only two loci of major effect are identified, but what is the evidence for linked modifiers?

> The major effect loci or switches are *ivory* and *optix*, which control melanisation and red/orange vs black, respectively, across wing and other cuticular surfaces. The modifiers are the narrower regions identified via GWAS, which are likely involved in *cis*-regulation of *ivory* and *optix*, and control where on the wing pigmentation changes occur. This is particularly evident in Figure 4A, where three distinct genomic intervals upstream of *optix* are associated with different aspects of orange/black patterning in *Mec. messenoides*. We have changed the wording and added a sentence to clarify this (lines 264-265).

7. In the second paragraph of the same section, the first sentence is a bit convoluted. Perhaps it should be broken into two sentences to more clearly convey the punchline.

> The sentence has been split as suggested (lines 276-277).

8. In this same paragraph, the authors claim that the limited number of paths might enable diverse taxa to join the mimicry ring more easily. But, I could argue that the limited and narrow pathway makes it very difficult! The key here is likely waiting time for mutations in these genes. In the future, it would be very interesting to know whether mutation rates at these loci are particularly high relative to the rest of the genome, as in the stickleback Pitx1 locus (Xie et al. 2019 Science).

> We have retained the sentence about the limited number of paths as it is worded as a speculation, rather than a “claim”. But we have now added a sentence speculating on the higher mutation rates at *ivory* and *optix* (lines 278-279).

9. Figure 2: why are there two regions of strong association at optix in Mel. marsaeus? Is this possibly an assembly error? A brief comment on this result would be welcome. Also, in the Figure 2 legend, please define ivory pro.

> We speculate that the two regions of association in *Mel. marsaeus* correspond to different correlated aspects of wing orange/black. This is similar to that seen in the *Mec. messenoides* GWAS plot that we further dissect in Figure 4A. An analogous analysis is not possible in *Mel. marsaeus* as the sequenced specimens do not show sufficient pattern variation. We have now added a sentence to this effect (lines 147-149).

*Ivory* pro is now defined in the Figure 2 legend.

10. Figure 4: I would appreciate pictures of the wild-type wings for comparison with the knockouts, especially for ivory, where the regions in which the gene have been deleted are not denoted. Also, in the legend for panel C, please indicate that the ivory expression is in white on the photo.

Thank you for the suggestion. We have changed this figure to include an image of the wild-type wing phenotype, and updated the figure legend to indicate the staining colour for *ivory* expression.

Reviewer #2: This manuscript documents convergent evolution of the genetic basis of convergent color patterns across highly divergent members of lepidopteran mimicry rings. The authors use genome-wide association studies to identify loci associated with color pattern in eight species, and find that the significant loci consistently include the genes ivory and optix. This suggests that the genetic mechanisms for transitions between the color phenotypes observed in this mimicry ring are highly constrained. The authors further demonstrate that even among closely related species, the shared genetic basis of color pattern is typically due to independent reuse of the same genes rather than introgression of the same mutations, and use Crispr-Cas9 knockouts and in situ hybridization to confirm the causal role of ivory and optix in one species.

This study is likely to be of broad interest due to the surprisingly high level of convergence in the genetics of color pattern. The authors are admirably thorough in addressing follow-up questions to the main result, the writing is clear and concise, and the methods and interpretations of results are appropriate. My only suggestion for improvement is to consider placing this study in a broader context of gene reuse, by noting other groups of organisms and other types of traits where similar patterns have been observed. In particular, color differences between and within vertebrate species, especially birds, often map to the same small set of genes. This might suggest that developmental pathways underlying coloration are more constrained than other traits frequently involved in local adaptation and/or species differences.

> We have now added a sentence to the end of the first paragraph of the “Repeatable and predictable evolution” section in line with the reviewer’s suggestion (lines 271-274).

Reviewer #3: This study addresses a deep and central question in evolutionary biology: What is the molecular basis of convergent phenotypes, and through what mechanisms do new forms arise?

A major novelty of this study is that it encompasses a very wide range of evolutionary timescales (1-120 Mya) to study the evolutionary genetic basis of convergent wing pattern polymorphisms. The striking finding is that alleles at the same two loci (ivory and optix) control equivalent wing pattern components across highly diverged species, including butterflies and a moth that form part of a complex of mimicry rings. The scale of the study is also impressive, requiring comparative whole genome analysis of multiple wing-pattern forms of seven species' population samples collected in the field. Functional validation of these loci is demonstrated for one of the species using CRISPR-Cas9 mosaic KO and in-situ hybridisation of developing wings.

Of particular interest and novelty is the discovery of a large inversion polymorphism in the moth (Chetone histrio) whose breakpoint junctions closely parallel a classic inversion polymorphism in Heliconius numata that is presumed to function as a supergene.

The other novelty of the study is that it finds no evidence that these alleles have been transferred horizontally via occasional inter-species hybridisation events, implying that they have arisen through de novo mutation many times. This suggests that the contrasting pattern observed within Heliconius may be restricted to closely related species.

The interpretation is appropriately cautious, recognising some limitations and outstanding questions.

I only have very minor presentational suggestions.

Main text, para 3: "..single butterfly species or genus" is followed by citations and sentences referring to two different genera
> The wording has been changed to clarify the meaning (line 80).

Main text, "Repeatable and predictable evolution" section: semi-colon after "dispute";

> Semicolon added as suggested (line 262).

the first sentence in the following paragraph doesn't quite make sense

> Also raised by Reviewer 1. The sentence has now been split and modified (lines 275-276).

Fig. 2. As the first narrative section relating to this figure is restricted to Ithomiini, would be useful to make it obvious which these are in the figure (e.g. vertical line)

> We now highlight the ithomiine species in this figure by labelling this clade in the figure.

Fig. 3. Light-blue shaded blocks would give greater visibility to significance colours and avoid confusion with the second use of purple

> In line with the suggestion, we have replaced the purple blocks with blue blocks in this figure.

**DATA RELATED REQUESTS**

(A) You may be aware of the PLOS Data Policy, which requires that all data be made available without restriction: <http://journals.plos.org/plosbiology/s/data-availability>. For more information, please also see this editorial: <http://dx.doi.org/10.1371/journal.pbio.1001797>

Note that we do not require all raw data. Rather, we ask that all individual quantitative observations that underlie the data summarized in the figures and results of your paper be made available in one of the following forms:

-Supplementary files (e.g., excel). Please ensure that all data files are uploaded as 'Supporting Information' and are invariably referred to (in the manuscript, figure legends, and the Description field when uploading your files) using the following format verbatim: S1 Data, S2 Data, etc. Multiple panels of a single or even several figures can be included as multiple sheets in one excel file that is saved using exactly the following convention: S1_Data.xlsx (using an underscore).

-Deposition in a publicly available repository. Please also provide the accession code or a reviewer link so that we may view your data before publication.

Regardless of the method selected, please ensure that you provide the individual numerical values that underlie the summary data displayed in the following figure panels as they are essential for readers to assess your analysis and to reproduce it:

Figure 1B, 2A-B, 3, 4A, S2, S3, S4, S5, S6, S7, S8, S9, S10, S11, S12, S13, S14, S15, S16A, S17C-F, S18C-F, S19C-F, S20C-E, S21C-F, S22C-D, S23C-D, S25, S29A-B, S30A-F, S31A-F, S32A-F, S36A-B, S38A-B

NOTE: the numerical data provided should include all replicates AND the way in which the plotted mean and errors were derived (it should not present only the mean/average values).

(B) Please also ensure that each of the relevant figure legends in your manuscript include information on *WHERE THE UNDERLYING DATA CAN BE FOUND*, and ensure your supplemental data file/s has a legend.

(C) Please note that we cannot accept sole deposition of code in GitHub, as this could be changed after publication. However, you can archive this version of your publicly available GitHub code to Zenodo. Once you do this, it will generate a DOI number, which you will need to provide in the Data Accessibility Statement (you are welcome to also provide the GitHub access information). See the process for doing this here: <https://docs.github.com/en/repositories/archiving-a-github-repository/referencing-and-citing-content>

> The DOI of the zenodo with the scripts is <https://doi.org/10.5281/zenodo.18762102>

> The scripts are accessible here: <https://zenodo.org/records/18762103>

(D) Please ensure that your Data Statement in the submission system accurately describes where your data can be found and is in final format, as it will be published as written there.

**TECHNICAL REQUESTS**

1) Please amend your detailed Financial Disclosure statement. This is published with the article. It must therefore be completed in full sentences and contain the exact wording you wish to be published.

1. Please clarify all sources of funding (financial or material support) for your study. List the grants (with grant number) or organizations (with url) that supported your study, including funding received from your institution.

2. State the initials, alongside each funding source, of each author to receive each grant.

3. State what role the funders took in the study. If the funders had no role in your study, please state: “The funders had no role in study design, data collection and analysis, decision to publish, or preparation of the manuscript.”

4. If any authors received a salary from any of your funders, please state which authors and which funders.

> Done

2) Please provide separate figure files in .tif or .eps format.

For more information about figure files please see our guidelines:

<https://journals.plos.org/plosbiology/s/figures>

<https://journals.plos.org/plosbiology/s/figures#loc-file-requirements>

3) We do not publish any copyright or trademark symbols that usually accompany proprietary names, eg (R), (C), or TM (e.g. next to drug or reagent names). Please remove all instances of trademark/copyright symbols throughout the text, including ® on page 10 and 11.

> Done

4) Please refer to the following article for PLOS Biology's best-practice recommendations for data presentation: <https://journals.plos.org/plosbiology/article?id=10.1371/journal.pbio.1002128>.

Guidelines for statistical reporting, if relevant, may be found here: <https://journals.plos.org/plosbiology/s/best-practices-in-research-reporting#loc-statistical-reporting>.

5) Some material included in your submission may be copyrighted. According to PLOS’s copyright policy, authors who use figures or other material (e.g., graphics, clipart, maps) from another author or copyright holder must demonstrate or obtain permission to publish this material under the Creative Commons Attribution 4.0 International (CC BY 4.0) License used by PLOS journals. Please closely review the details of PLOS’s copyright requirements here: PLOS Licenses and Copyright. If you need to request permissions from a copyright holder, you may use PLOS's Copyright Content Permission form.

Please respond directly to this email and provide any known details concerning your material's license terms and permissions required for reuse, even if you have not yet obtained copyright permissions or are unsure of your material's copyright compatibility. Once you have responded and addressed all other outstanding technical requirements, you may resubmit your manuscript within Editorial Manager.

Potential Copyright Issues:

i) Fig S14C, S17B, S18B, S19, S20, S21, S22 , S23, S26, S27, S28, S33, S34, S35, S36: Please confirm (a) that you are the photographer; or (b) provide written permission from the photographer to publish the photo(s) under our CC BY 4.0 license.

> These photographs were provided by the authors.

ii) Fig 1, 2, S1, S2, S10, S14D, S17E, S17F, S18, S19, S20, S21, S22 , S23, S37B: Please confirm whether you drew the images / clip-art within the figure panels by hand. If you did not draw the images, please provide (a) a link to the source of the images or icons and their license / terms of use; or (b) written permission from the copyright holder to publish the images or icons under our CC BY 4.0 license. Alternatively, you may replace the images with open source alternatives. See these open source resources you may use to replace images / clip-art:

- [https://commons.wikimedia.org](https://commons.wikimedia.org/)

- <https://openclipart.org/>

> “Clip-art” butterfly images were drawn by E.S.M.v.d.H., based on our own butterfly photographs.

iii) Fig 1, S1, S17A, S18A, S19, S20 , S21, S22 : please (a) provide a direct link to the base layer of the map (i.e., the country or region border shape) and ensure this is also included in the figure legend; and (b) provide a link to the terms of use / license information for the base layer image or shapefile. We cannot publish proprietary or copyrighted maps (e.g. Google Maps, Mapquest) and the terms of use for your map base layer must be compatible with our CC BY 4.0 license.

Note: if you created the map in a software program like R or ArcGIS, please locate and indicate the source of the basemap shapefile onto which data has been plotted.

If your map was obtained from a copyrighted source please amend the figure so that the base map used is from an openly available source. Alternatively, please provide explicit written permission from the copyright holder granting you the right to publish the material under our CC BY 4.0 license.

If you are unsure whether you can use a map or not, please do reach out and we will be able to help you. The following websites are good examples of where you can source open access or public domain maps:

* U.S. Geological Survey (USGS) - All maps are in the public domain. ([http://www.usgs.gov](http://www.usgs.gov/))

* PlaniGlobe - All maps are published under a Creative Commons license so please cite “PlaniGlobe, [http://www.planiglobe.com](http://www.planiglobe.com/), CC BY 2.0” in the image credit after the caption. (<http://www.planiglobe.com/?lang=enl>)

* Natural Earth - All maps are public domain. (<http://www.naturalearthdata.com/about/terms-of-use/>)

> The map sources are now added to the figure legends. We used:

<http://www.naturalearthdata.com/about/terms-of-use/>

<https://docs.stadiamaps.com/limits/>
